# A New Interval Belief Rule Base Model Based on Hybrid Optimization and Adaptive Reference Intervals for Diesel Engine Health State Assessment

**DOI:** 10.3390/s26082342

**Published:** 2026-04-10

**Authors:** Hongming Zheng, Bing Xu, Motong Zhao, Hongyao Du, Wei He

**Affiliations:** 1 School of Computer Science and Information Engineering, Harbin Normal University, Harbin 150025, China; 2024300699@stu.hrbnu.edu.cn (H.Z.); 18845127666@163.com (H.D.); 2 School of Integrated Circuits, Dalian University of Technology, Dalian 116081, China; 20232261054@dlut.edu.cn

**Keywords:** health state assessment, adaptive reference intervals, interval belief rule base, hybrid optimization

## Abstract

As the core power unit of complex electromechanical systems, accurate health assessment of diesel engines is essential for safe operation. The Interval Belief Rule Base (IBRB) method integrates observed data with expert knowledge to support system assessment. However, engine operating parameters change over time because of wear and aging. Additionally, traditional optimization methods struggle to balance global search speed with local convergence efficiency. To address these issues, this paper proposes an Interval Belief Rule Base method based on Hybrid Optimization and Adaptive Intervals (IBRB-HOAI). First, an adaptive reference interval is introduced by combining K-means clustering and quantile interval estimation, dynamically generated based on the actual operating state of the engine. The health assessment baseline is optimized. The applicability of the model is enhanced. Second, the global exploration ability of particle swarm optimization is combined with the local refinement ability of the projected covariance matrix adaptation evolution strategy. The model parameters are collaboratively optimized. Finally, experimental verification is conducted on a diesel engine dataset containing 2700 sample points. Compared with the traditional IBRB method, the proposed method achieves a significant reduction in MSE of 97.5%. It outperforms other machine learning methods. The effectiveness of the proposed method is verified.

## 1. Introduction

As the core power unit of complex electromechanical systems, the health status of diesel engines directly affects the reliability, safety, and economic efficiency of the entire system. If a diesel engine fails due of performance degradation or sudden malfunction, it may lead to significant economic losses or even serious safety incidents. Therefore, conducting accurate and timely assessments of the health status of diesel engines and implementing predictive maintenance based on these assessments are of vital significance for ensuring the continuous and stable operation of critical systems [1,2,3,4].

Currently, methods for Health Status Assessment (HSA) are categorized into three primary types [5,6]: knowledge-based assessment methods, data-driven assessment methods, and semi-quantitative assessment methods based on information fusion. Knowledge-based methods rely heavily on expert knowledge and inference frameworks. Owing to their ability to clearly explain the decision-making process, these methods excel in providing interpretable and transparent assessment results. For example, Badida et al. utilized Fuzzy Fault Tree Analysis (FFTA) for the health status assessment of oil and gas pipelines [7]. They analyzed the probability of pipeline failure by integrating FFTA with expert elicitation. Zhang and Meng employed a probabilistic ship domain to capture the diversity of navigation behaviors, enabling the assessment of ship collision risk [8]. Yazdi et al. proposed an uncertainty-handling scheme based on Fault Tree Analysis (FTA) for health status assessment, which can be applied to evaluate the health condition of flywheels [9]. These knowledge-driven approaches enable the full integration and utilization of existing knowledge, including model algorithms, practical experience, and multidisciplinary expertise. Most of these methods are underpinned by a reliable and comprehensive theoretical framework. However, owing to the complexity of the environment and system structure, various uncertainties are introduced when models are constructed based on expert knowledge. The precision of these models is often inadequate. As a result, the accuracy of knowledge-driven HSA models cannot meet these requirements. Moreover, such models are unsuitable for assessing the health status of engines.

On the other hand, data-driven methods are utilized to assess system safety and performance through the analysis of condition monitoring data. Owing to their objectivity and accuracy, these methods have gained widespread attention [10], with models such as artificial neural networks and support vector machines being extensively applied. For example, Adhikari et al. proposed an analytical framework based on recovery risk analysis to assess the operational health of bulk power systems integrated with wind power following major disturbance events [11]. AL-Masri et al. proposed an adaptive artificial neural network-based method to enhance power system stability and predict generator rotor angles [12]. In the field of fault diagnosis, convolutional neural networks (CNNs) are widely used to automatically extract time-frequency domain features from vibration signals, enabling end-to-end fault identification [13,14]. Long short-term memory (LSTM) networks, with their ability to capture temporal dependencies, have been successfully applied to remaining useful life prediction and fault trend analysis [15,16]. In recent years, Transformer-based methods have demonstrated excellent performance in fault diagnosis because of their powerful global feature extraction capability [17]. Roy et al. integrated LSTM networks and feedforward neural networks (FFNNs) with the backpropagation algorithm (BPA) to precisely locate faults in grid-connected microgrid systems [18]. However, although these methods are powerful, their effectiveness depends largely on the availability of fault data. With the increasing stability and reliability of systems, the frequency of failures is reduced, making it difficult to collect sufficient data for accurate modeling. As neural networks are black-box models, their interpretability is generally poor, and the decision-making process is difficult to understand. This may limit the degree of trust in and acceptance of the assessment results. In recent years, significant progress has been made in explainable artificial intelligence. A range of mature interpretability techniques have been widely applied to analyze the decision-making processes of complex models. For example, local explanation methods such as SHAP and LIME are used to quantify the contribution of each input feature to the model output [19]. In this way, neural networks are provided with interpretability at the level of individual predictions. Attention mechanisms are employed to visualize the input regions focused on by the model [20]. Effective visualization of decision-making processes has thus been achieved in areas such as natural language processing and fault diagnosis. In addition, a relatively complete theoretical framework has been established for interpretability research on deep learning models [21]. With the development of these techniques, neural network models are now able to maintain high accuracy, but their decision-making processes are significantly more understandable and trustworthy [22]. Therefore, it is no longer justified to dismiss the value of neural networks in health status assessment simply by referring to them as “black boxes,” given the current state of technological progress. A more fundamental challenge for data-driven methods lies in their heavy reliance on the availability of fault data. As system stability and reliability improve, the frequency of faults decreases. Consequently, sufficient fault data are often difficult to collect for accurate modeling. This constitutes the core obstacle to the engineering application of data-driven methods.

To address the limitations of both knowledge-based and data-driven methods, semi-quantitative approaches offer a balanced alternative [23]. For example, Chang et al. proposed a health status assessment method for the operation of maritime autonomous surface ships [24]. This method integrates failure mode and effects analysis (FMEA) with Bayesian networks (BN) and evidential reasoning (ER) to quantify the identified levels of health status. Zhang et al. developed a two-layer Monte Carlo sampling-based model for FTA of failures in airborne refrigeration systems [25]. Huang et al. proposed a hybrid that combines prior bootstrapping techniques with data-driven approaches to provide accurate and reasonable wind power prediction results [26]. Additionally, methods such as the analytic hierarchy process (AHP) [27], Monte Carlo simulation [28], and belief rule base (BRB) have been employed.

The IBRB extends the traditional BRB by introducing interval-based representations [29,30]. Its core characteristic is a significantly enhanced ability to express and handle uncertainties. Specifically, it allows antecedent attribute reference values and rule confidence degrees to be expressed in interval form. This better aligns with the inherent vagueness of expert knowledge and provides greater robustness against noisy data. Furthermore, the output results inherently contain measures of uncertainty. This makes it particularly suitable for complex scenarios such as diesel engine fault diagnosis and health status assessment [31,32]. It effectively handles gradual state transitions during performance degradation and addresses issues of incomplete information. Despite its many advantages, the IBRB still faces challenges in practical applications [33]. First, the issue of fixed input reference intervals. In the traditional IBRB model, the reference intervals of prerequisite attributes are usually set based on expert experience under initial operating conditions. These intervals remain unchanged after the model is constructed. However, during the long-term operation of a diesel engine, factors such as wear, carbon deposition, and changes in clearance cause the reasonable ranges of key operating parameters to shift dynamically. Fixed reference intervals are unable to reflect this physical degradation process. As a result, evaluation bias is introduced in the later stages of the engine lifecycle. Second, an imbalance between global and local search capabilities in parameter optimization algorithms. Parameter optimization of the IBRB model is a typical nonlinear, multi-constraint optimization problem. In existing research, a single optimization algorithm, such as particle swarm optimization or differential evolution, is often used. However, when these algorithms are applied to the high-dimensional parameter space of the IBRB, a contradiction is faced between the global search capability and local convergence efficiency. Algorithms with strong global search capability converge slowly and suffer from insufficient accuracy in later stages. Algorithms with strong local search capability are prone to becoming trapped in local optima, making it difficult to find the globally optimal parameter combination. Third, from the perspective of data acquisition. As highly reliable equipment, diesel engines accumulate very limited fault data under normal operating conditions. Data-driven methods require numerous labeled fault samples for training, which is difficult to satisfy in engineering practice. Knowledge-based methods, while not reliant on fault data, suffer from ambiguity, inconsistency, and incompleteness of expert knowledge. Consequently, models built solely on knowledge are constrained in accuracy. To address the above limitations of the IBRB model and existing engine diagnostic methods, this paper proposes an interval belief rule base model based on hybrid optimization and adaptive reference intervals, referred to as IBRB-HOAI. This model is referred to as IBRB-HOAI. This method incorporates an adaptive reference interval update mechanism, moving away from traditional fixed reference intervals and enabling dynamic adjustment of the input reference intervals in the IBRB. Furthermore, a hybrid optimization algorithm, namely, PSO and P-CMA-ES is proposed for parameter training in the IBRB [34]. The hybrid algorithm aims to synergize the global rapid exploration capability of the PSO with the powerful local precision exploitation ability of the P-CMA-ES. This approach overcomes the limitations of traditional optimization algorithms, enabling efficient and high-precision identification of the optimal parameter set for the IBRB and significantly enhancing the model’s assessment performance. The main contributions of this paper are outlined as follows:

The paper is structured as follows: Section 1 analyzes the current status of health assessment research and proposes the IBRB-HOAI model for diesel engines. Section 2 discusses the inherent limitations of IBRB and the challenges resolved in constructing the IBRB-HOAI model. Section 3 provides a systematic introduction to the new model from three perspectives: model construction, inference, and optimization. Section 4 validates the model’s performance through simulation experiments and a comparative analysis on diesel engines. Section 5 summarizes the research findings and outlines future research directions.

## 2. Problem Description

To construct a diesel engine health status assessment model based on the IBRB-HOAI, the following two problems need to be addressed.

Problem 1: How to address the changes in engine health status caused by factors such as wear and aging.

The normal operational parameters of an engine vary under different conditions. Normal parameters of the engine are altered under varying operating conditions. The health status of the engine is consequently affected. The prediction accuracy is also influenced. An adaptive reference interval is constructed by integrating K-means clustering with quantile estimation. Healthy benchmark ranges for performance parameters are then dynamically calculated and updated using this interval.

(1) $$RI_{S}=Q(K\left(X)\right)$$

where $X={\left[x_{1},\cdots ,x_{S}\right]}^{T}$ is defined as the input attribute, and the feature dimension is represented by S. Each sample contains S monitoring attributes. A K-means clustering function is denoted as K(•), partitions the data into several clusters. The cluster with the largest sample size and the most compact distribution is selected as the healthy reference subset. A quantile estimation function is denoted as Q(•), calculates reference intervals based on the empirical distribution of each feature in the healthy subset. The adaptive reference interval for the sth feature is denoted as $RI_{S}$.

Problem 2: How to address the issues of optimization algorithms becoming trapped in local optima and their relatively slow convergence speed in the early stages.

When solving complex global optimization problems using traditional optimization algorithms, the ability of the PSO algorithm to maintain population diversity in later stages is lacking. This prevents the avoidance of premature convergence. Moreover, the P-CMA-ES has a relatively low initial search speed. Consequently, the optimization model fails to achieve optimal results. A hybrid optimization strategy is designed as follows:

(2) $$\Phi_{best}=H_{PSO-PCMAES}(\Phi ,MSE,\Omega )$$

where the hybrid optimization operator is defined as $H_{PSO-PCMAES}(•)$. It is formed by combining PSO and P-CMA-ES. This operator is proposed in this paper. The initial parameter set is referred to as Φ. It is determined based on expert knowledge. The optimization objective function is defined as MSE. The set of algorithm hyperparameters is referred to as Ω. This set includes the population size, number of iterations, convergence threshold, and others. The optimal BRB model parameter set is denoted as $\Phi_{best}$. It is obtained through optimization.

## 3. Construction of IBRB-HOAI

On the basis of the aforementioned issues, particularly the complex and variable operating states of diesel engines and the inability of traditional assessment methods to adapt dynamically, this study proposes an IBRB-HOAI model for more accurate and robust HSA.

**Figure 1:**
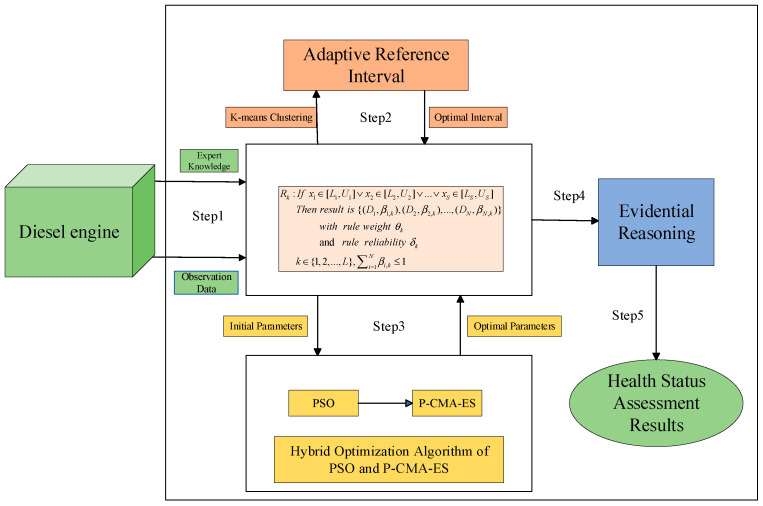
The IBRB-HOAI model’s overall structure.

This model integrates three key components into a comprehensive assessment framework: the construction of adaptive reference intervals, the modeling and inference process of the IBRB, and the hybrid optimization strategy. The overall framework of the constructed health status assessment model is illustrated in Figure 1. The input data for the IBRB-HOAI model include monitoring data used to define initial belief rules, reference intervals, and expert knowledge. By integrating these elements, a comprehensive health status assessment can be achieved. Adaptive reference interval optimization is performed to obtain the optimal reference intervals. Model accuracy and complexity are considered by using a hybrid optimization algorithm based on PSO and P-CMA-ES. The output is the predicted system health status [35].

In this study a framework for diesel engine health state assessment based on an IBRB-HOAI model is proposed. The core process, as illustrated in Figure 1, comprises five key steps:

Step 1: Construction of the Expert Knowledge Rule Base

First, based on prior knowledge, an expert knowledge rule base is constructed. Each rule $R_{k}$ is formally expressed as follows: if $\;x_{1}\in [L_{1},U_{1}]\vee x_{2}\in [L_{2},U_{2}]\vee \ldots \vee x_{S}\in [L_{S},U_{S}]$ then the output is a belief distribution $\left\{(D_{1},\beta_{1,k}),(D_{2},\beta_{2,k}),\ldots ,(D_{N},\beta_{N,k})\right\}$.

Step 2: Construction of Adaptive Reference Intervals

Based on diesel engine operational data, feature parameters are first clustered using a method that combines K-means clustering with quantile analysis. An adaptive stepwise optimization is then applied to determine the reference intervals for each parameter, providing a dynamic and adaptive threshold basis for subsequent rule construction.

Step 3: Initialization of Parameters for Hybrid Optimization Algorithms

This step adopts a hybrid strategy for parameter initialization: on the one hand, the global search capability of the PSO algorithm is leveraged to obtain preliminary solutions; on the other hand, the local fine-tuning capability of the P-CMA-ES is incorporated to refine the solutions. Through synergistic collaboration, high-quality initial parameters are generated, laying a foundation for subsequent in-depth optimization.

Step 4: Evidential Reasoning

When new observation data are input, this step employs the ER method for information fusion and decision-making. This approach can effectively handle uncertainties and conflicting information generated during rule-based inference, synthesizing the belief distributions output by multiple activated rules.

Step 5: Health State Assessment

The integrated belief degrees for each health state level of the diesel engine are ultimately calculated, yielding an intuitive and reliable health state assessment result, thereby providing decision support for condition-based maintenance.

### 3.1. Adaptive Reference Interval Construction

The accuracy of diesel engine health assessment heavily relies on a reasonable evaluation benchmark, i.e., the reference intervals that define the normal operating range of each monitoring feature. Traditional fixed-threshold methods often fail to accommodate the dynamic variations caused by engine wear, aging, and changing operating conditions. To address this, we propose an adaptive reference interval construction method that combines K-means clustering with quantile-based estimation [36]. This approach automatically extracts a “healthy” reference subset from raw historical data and then builds statistically robust intervals.

Step 1: K-means clustering partitions the data

Let the historical dataset be denoted as $X={\left[x_{1},\cdots ,x_{S}\right]}^{T}$ where each sample $x_{i}\in R^{S}$ consists of S monitoring attributes (e.g., vibration amplitudes, temperatures). K-means clustering partitions the data into K disjoint clusters $C=\{C_{1},C_{2},\ldots C_{K}\}$ by minimizing the within-cluster sum of squares (WCSS):

(3) $$J\left(C,\zeta \right)=\sum_{k=1}^{K}\sum_{x_{i}\in C_{k}}{\left\|x_{i}-\zeta_{k}\right\|}^{2}$$

where $\zeta_{k}$ is the centroid of cluster $C_{k}$. The centroids are iteratively updated as:

(4) $$\zeta_{k}^{\left(t+1\right)}=\frac{1}{\left|C_{k}^{\left(t\right)}\right|}\sum_{x_{i}\in C_{k}^{\left(t\right)}}x_{i}$$

The underlying assumption is that samples corresponding to the engine’s normal state are statistically similar and thus form a compact cluster in the feature space. In practice, we hypothesize that the healthy state is the most prevalent operating condition; therefore, the largest cluster is selected as the reference cluster representing the healthy state:

(5) $$C_{\mathrm{normal}}=\arg\max_{k}\left|C_{k}\right|$$

Step 2: Constructing Reference Intervals Using Quantiles

Once $C_{\mathrm{normal}}$ is identified, we extract the values of each feature from all samples within this cluster. For the sth feature (s=1,…,S), let $X_{S}^{\mathrm{ref}}=\left\{x_{S,i}|x_{i}\in C_{\mathrm{normal}}\right\}$ denote the set of observed values. Instead of directly using the centroid, we employ the quantile method to construct a reference interval that captures the natural spread of healthy data while being robust to outliers. The adaptive reference interval for feature s is defined as:

(6) $$RI_{S}=[L_{S},U_{S}]=[Q_{\alpha /2}(X_{S}^{ref}),Q_{1-\alpha /2}(X_{S}^{ref})]$$

where the pth quantile function is denoted by $Q_{p}(•)$, and α is a significance level (typically α=0.05, yielding a 95% reference interval). This interval indicates that under healthy conditions, the feature value is expected to lie within $\left[L_{S},U_{S}\right]$ with high probability.

Relation between K-means and Quantile Estimation: The K-means algorithm serves solely to segment the data and isolate a subset that is likely healthy. It does not directly produce intervals; rather, it provides the sample set $X_{S}^{\mathrm{ref}}$ from which statistical intervals are derived. The centroids themselves are not used as interval boundaries—they merely guide the cluster assignment. This two-stage process ensures that the reference intervals are data-driven and reflect the actual distribution of healthy measurements, rather than being fixed a priori.

K-means is a classic unsupervised clustering algorithm. It can partition samples into several clusters based on their intrinsic similarity. In health assessment scenarios, the healthy state is often the primary operating mode. It corresponds to the most compact cluster in the feature space. This cluster also typically has the largest sample size. By maximizing the intra-cluster similarity, the “healthy reference cluster” can be automatically separated from the raw data. These raw data are a mixture of healthy and faulty samples. No manual labeling or prior knowledge is required for this separation. Subjective screening bias is therefore avoided. The selection of reference samples is based entirely on the data distribution. Strong objectivity and reproducibility are achieved through this process.

If the cluster centroid is used directly as the reference boundary, distribution information is lost. This approach is also susceptible to interference from outliers. With the quantile method, the empirical distribution quantiles are extracted. These quantiles are derived from each feature within the healthy cluster. The central tendency and the range of fluctuation can be effectively characterized. Resistance to outliers is an inherent property of this method. A 95% reference interval is adopted. It has a clear probabilistic interpretation in statistics. The rationality of the interval boundaries is ensured.

The combination of K-means and quantiles forms an adaptive two-stage framework. First, the “healthy reference subset” is dynamically identified. This identification is performed by clustering under the current observation environment. Then, the reference intervals are generated. They are based on the statistical characteristics of this subset. When the engine operating state changes, this process can be re-executed. The intervals are updated automatically. The evaluation benchmark is thus kept consistent with the latest data characteristics. This adaptability significantly improves the generalization ability of the model. This improvement is especially notable in complex situations.

### 3.2. Modeling of the IBRB-HOAI Model

The assessment of diesel engine health status is the focus of this study. As typical examples of complex electromechanical systems, the operational status of diesel engines directly affects the safety, reliability, and economic efficiency of the entire equipment system. To assess the health status of diesel engines accurately [37], promptly, and quantitatively, a health assessment model is constructed in this study. This model integrates a hybrid optimization algorithm strategy with an IBRB featuring adaptive reference intervals.

The IBRB-HOAI is established via optimized parameters. The final evaluation result is output as follows:

(7) $$\begin{array}{c} R_{k}:If\;x_{1}\in [L_{1},U_{1}]\vee x_{2}\in [L_{2},U_{2}]\vee \ldots \vee x_{S}\in [L_{S},U_{S}]\\ Then\;result\;is\;\{(D_{1},\beta_{1,k}),(D_{2},\beta_{2,k}),\ldots ,(D_{N},\beta_{N,k})\}\\ with\;rule\;weight\;\theta_{k}\\ \;\mathrm{and}\;rule\;reliability\;\delta_{k}\;\\ k\in \{1,2,\ldots ,L\},\sum_{i=1}^{N}\beta_{i,k}\leq 1\; \end{array}$$

where the input premise attributes are denoted by $x_{i}(i=1,2,\ldots ,S)$ in this model and S is the number of premise attributes. The output result is denoted by ${(D}_{i},\beta_{i,k})(i=1,2,\ldots ,N)$. The rule weight is indicated by $\theta_{k}$ and the rule reliability is expressed as $\delta_{k}$. The adaptive reference intervals are characterized by $\left[L_{S},U_{S}\right]$.

### 3.3. Inference of the IBRB-HOAI Model

#### 3.3.1. Belief Rules Activation

In IBRB, a rule is activated according to the consistency level of its required attributes. Specifically, a rule is activated only when the matching degrees of all premise attributes are non-zero. The premise attribute calculation in IBRB is as follows:

(8) $$a_{i}^{k}=\left\{\begin{array}{ll} \frac{A_{i}^{l+1}-x_{i}}{A_{i}^{l+1}-A_{i}^{l}} & k\;=\;l,\;A_{i}^{l}\leq x_{i}\leq A_{i}^{l+1}\\ 1-a_{i}^{k} & k\;=\;l\;+\;1\\ 0 & \begin{array}{l} k=1\cdots L,k\neq l,l+1 \end{array} \end{array}\right.$$

where the matching degree between the ith attribute and the kth rule is given by $a_{i}^{k}$. The sample data for the ith attribute are denoted by $x_{i}$. The reference values for this attribute in the lth rule are represented by the set $A_{i}^{l}$.

In contrast to the traditional BRB, the IBRB-HOAI model necessitates the consideration of rule activation intervals. The activation of an associated rule is contingent upon the data sample of a prerequisite attribute falling within a defined interval. As illustrated in Table 1, when the two antecedent attributes fall within the intervals $\left[a_{2},b_{2}\right]$ and $\left[c_{1},d_{1}\right]$, the second and fifth rules are activated accordingly.

#### 3.3.2. Inference Using the ER Rule

Intervals in rule activation must be accounted for by the IBRB-HOAI model. The associated rule is activated when the data sample of a prerequisite attribute is found within the defined interval. The ER rule is incorporated into the inference process of the IBRB-HOAI model. Rule reliability is prioritized by this incorporation. The completeness of the model’s inference process is enhanced by the introduction of the ER rule. Its scalability is also improved by this introduction. To some extent, the rules in the IBRB-HOAI model correspond to the evidence in the ER rule. The inference process of the IBRB-HOAI model is described as follows.

After the input data are preprocessed, the ER rule is applied for evidence fusion. The reliability of evidence is affected by differences in collection methods and noise interference. The ER rule fully accounts for the reliability of evidence, addressing the challenge in IBRB inference where evidence reliability is not considered. The evidence $e_{i}$ can be represented as the following belief distribution.

(9) $$e_{i}=\{(D_{n},\beta_{n,i}),n=1,\ldots ,N;(\Theta ,\beta_{\Theta ,i})\;|\;0\leq \beta_{n,i}\leq 1,\;\sum_{n=1}^{N}\beta_{n,i}\leq 1\}$$

where the identification framework is denoted by $\Theta =\{D_{1},\ldots ,D_{N}\}$ in this framework. The degree of belief that the result $D_{n}$ is expressed as $\beta_{n,i}$. The global ignorance regarding the attribute of Θ is characterized by $\beta_{\Theta ,i}$. The weight of the evidence is assigned as $\theta_{i}(i=1,\ldots ,L)$, while the reliability of the evidence is represented by $\delta_{i}(i=1,\ldots ,L)$, both of which satisfy $\theta_{i}\in [0,1]$ $\delta_{i}\in [0,1]$. After mixing and weighting the evidence weight and evidence reliability, the belief distribution can be expressed as follows:

(10) $$m_{i}=\{(D_{n},{\tilde{m}}_{n,i}),\forall D_{n}\subseteq \Theta ;(\beta (\Theta ),{\tilde{m}}_{\beta (\Theta ),i})\}$$

where the power set is denoted by β(Θ). The mixed probability mass of evidence i at the evaluation level $D_{n}$ is represented as ${\tilde{m}}_{n,i}$

(11) $${\tilde{m}}_{n,i}=\left\{\begin{array}{ll} 0, & D_{n}=\emptyset \\ c_{\gamma w,i}m_{n,i}, & D_{n}\subseteq \Theta ,D_{n}\neq \emptyset \\ c_{\gamma w,i}(1-\delta_{i}), & D_{n}=\beta (\Theta ) \end{array}\right.$$

(12) $$c_{\gamma w,i}=1/(1+\theta_{i}-\delta_{i})$$

(13) $$m_{n,i}=\theta_{i}\beta_{n,i}$$

where a normalization coefficient is defined as $c_{\gamma w,i}$. This coefficient must satisfy the condition $\sum_{n=1}^{N}{\tilde{m}}_{n,i}+{\tilde{m}}_{\beta (\Theta ),i}=1$. For L independent pieces of evidence, the combined support degree $\beta_{n,e(L)}$ can be calculated as follows.

(14) $$\forall D_{n}\in \Theta ,{\hat{m}}_{n,e(k)}=[(1-\delta_{k})m_{n,e(k-1)}+m_{\beta (\Theta ),e(k-1)}m_{n,k}]+\sum_{A\cap B=D_{n}}m_{A,e(k-1)}m_{B,k}$$

(15) $${\hat{m}}_{\beta (\Theta ),e(k)=}(1-\delta_{k})m_{\beta (\Theta ),e(k-1)}$$

(16) $$m_{n,e(k)}=\left\{\begin{array}{ll} 0, & D_{n}=\emptyset \\ \frac{{\hat{m}}_{n,e(k)}}{\sum_{A\subseteq \Theta }{\hat{m}}_{A,e(k)}+{\hat{m}}_{\beta (\Theta ),e(k)}}, & D_{n}\neq \emptyset \end{array}\right.$$

(17) $$\beta_{n,e(k)}=\left\{\begin{array}{ll} 0, & D_{n}=\emptyset \\ \frac{{\hat{m}}_{n,e(k)}}{\sum_{A\subseteq \Theta }{\hat{m}}_{A,e(k)}}, & D_{n}\subseteq \Theta ,D_{n}\neq \emptyset \end{array}\right.$$

where k=1,2,…L. After k merged the first pieces of evidence, the confidence level for grade $D_{n}$ was recorded as $\beta_{n,e(k)}$. This satisfies both $m_{n,e(1)}=m_{n,1}$ and $m_{\beta (\Theta ),e(1)}=m_{\beta (\Theta ),1}$.

The following results are obtained from the above reasoning process: the output belief distribution and the output expected utility value.

(18) $$e(L)=\{(D_{n},\beta_{n,e(L)}),n=1,\ldots ,N,(\Theta ,\beta_{\Theta ,e(L)})\}$$

(19) $$y=\sum_{n=1}^{N}u(D_{n})\beta_{n,e(L)}+u(\Theta )\beta_{\Theta ,e(L)}$$

where the final expected utility value is represented by y. The final expected utility value. The utility of $D_{n}$ is denoted by $u(D_{n})$.

### 3.4. Optimization of the IBRB-HOAI Model

The optimization process of the IBRB-HOAI model is nonlinear. A hybrid optimization algorithm combining PSO and P-CMA-ES is employed in the training of the IBRB-HOAI model. Complementary mechanisms are utilized to achieve efficient global optimization. The core advantage of the PSO algorithm lies in its efficient global exploration capability. Particle velocities and positions are updated based on social collaboration and self-cognition. Extensive traversal of the solution space is realized by particles tracking individual optima and global optima. No gradient information is relied upon. Strong adaptability is therefore exhibited. Rapid escape from local optima is enabled. Potential optimal regions within the solution space are explored. However, an inherent limitation of the PSO is observed. As the number of iterations increases, the particle velocities gradually converge. Insufficient local convergence accuracy tends to occur. Particularly in local regions near the global optimum, the update step size of particles cannot be adaptively adjusted. Slow convergence speed and suboptimal solution accuracy are consequently caused.

The P-CMA-ES algorithm is an improved version of the CMA-ES. Its core advantage is superior local refinement capability. The covariance matrix is adaptively adjusted. The search step size and direction are dynamically optimized according to the local characteristics of the solution space. High-precision convergence is achieved within local regions. This algorithm is especially suitable for fine-grained search near the optimal solution in continuous optimization problems. Nonlinear parameter constraints within the belief rule base are effectively handled by this algorithm. Through dynamic adjustment of the search distribution and constraint projection, solution feasibility is ensured. Convergence speed and stability in complex BRB structure optimization are significantly improved. Nevertheless, a limitation of P-CMA-ES is recognized. Global exploration capability is lacking in the initial search stage. Premature convergence to local optima within the initial region is likely. In high-dimensional solution spaces, search efficiency in the initial stage is considerably lower than that of the PSO.

Based on the above characteristics, the PSO + P-CMA-ES hybrid strategy is designed. The complementary advantages of the two algorithms are achieved through a “PSO-first global exploration, P-CMA-ES-second local refinement” scheme. Inherent limitations of each algorithm are thereby avoided. Rapid traversal of the solution space is performed by the PSO. The approximate region containing the global optimum is located. High-precision search within this region is then conducted by P-CMA-ES. The accuracy of the final solution is thus enhanced.

The hybridization of PSO and P-CMA-ES is justified not only by empirical results but also by clear theoretical motivations. Single optimization algorithms face inherent trade-offs when handling high-dimensional, nonlinear, and constrained belief rule base optimization problems: PSO exhibits strong global exploration but suffers from low local convergence accuracy, while P-CMA-ES offers superior local refinement but lacks global search capability in the initial stage. PSO enables broad traversal of the solution space without gradient information, quickly locating the approximate region of the global optimum. However, its convergence speed near the optimum is slow, and its step size cannot be adaptively adjusted. In contrast, P-CMA-ES adaptively adjusts the covariance matrix and step size, achieving high-precision convergence in local regions, but it is prone to premature convergence to local optima in the early stage due to the lack of global guidance.

Thus, the theoretical advantage of the hybrid strategy lies in the complementary mechanism: PSO compensates for the initial blindness of P-CMA-ES through global exploration, while P-CMA-ES compensates for the local inaccuracy of PSO through high-precision refinement. This “global-first, local-second” scheme is theoretically expected to improve both global optimality and local accuracy without sacrificing search efficiency, outperforming either algorithm alone in complex BRB optimization.

To ensure the reproducibility of our method, this section provides a complete description of the configuration of the proposed hybrid optimization framework. The hyperparameters of the optimization algorithm are presented in Table 2.

This framework adopts a sequential ensemble approach. First, the PSO phase is executed to search for promising regions globally. After the PSO phase concludes, its optimal solution is used as the initial mean vector for the PCMA-ES module. Subsequently, PCMA-ES takes over the optimization process to perform fine-grained local search near the current optimal solution. This design combines the global exploration capability of PSO with the efficient local convergence property of PCMA-ES.

This framework employs a fixed serial coordination mechanism. The specific rules are shown in Table 3 below.

Traditional health state assessment methods are limited by expert knowledge. The initial model parameters and structures require further adjustment based on observed samples. The hybrid optimization process using the PSO and P-CMA-ES algorithms is introduced below. For the IBRB-HOAI-based health state assessment model, the optimization objective is to ensure that the estimated health state value is as close as possible to the actual value. During the optimization process of the IBRB-HOAI model, the objective function is defined as the mean squared error (MSE) between the health status estimated by the model and the actual health status. This metric is used to measure the fitting accuracy of the model. It is minimized as the objective during optimization. The optimization model is constructed as follows:

(20) $$\begin{array}{l} \min\;MSE(\theta^{*},\beta^{*},\delta^{*})\\ \min\;MSE(•)\\ s.t.0\leq \beta_{i,k}^{*}\leq 1,n=1,2,\ldots ,N,k=1,2,\ldots ,L\;\;\\ \sum_{i=1}^{N}\beta_{i,k}^{*}=1,k=1,2,\ldots ,L\;\;\\ 0\leq \theta_{k}^{*}\leq 1,k=1,2,\ldots ,L\\ 0\leq \delta_{k}^{*}\leq 1,k=1,2,\ldots ,L \end{array}$$

where the nth belief degree in the kth rule is represented by $\beta_{n,k}^{*}$ and the weight of the kth rule is denoted by $\theta_{k}^{*}$. The rule reliability parameter is defined as $\delta_{k}^{*}$.

The optimized parameters include the rule weight, belief degree, and rule reliability. The optimization model is divided into two parts: parameter optimization and structure adjustment. When the belief degree of a belief rule falls below the threshold, its weight is adjusted to zero. The rule no longer participates in training. This simplifies the model and better reflects the actual situation. This allows the structure to be adjusted for complex systems. Model complexity is thereby reduced. Additionally, the parameters of the IBRB-HOAI model must possess physical meaning. Training must be conducted under constraints.

The mean squared error (MSE) between the estimated health state and the actual health state indicates the accuracy of the health assessment model:

(21) $$\mathrm{MSE}(\theta^{*},\beta^{*},\delta^{*})=\frac{1}{H}\sum_{i=1}^{H}{\left(y_{i}-y_{i}^{actual}\right)}^{2}$$

where the actual health state is represented by $y_{i}^{actual}$ and the estimated output of the health state assessment model is denoted by $y_{i}$. The total number of training samples is defined as H.

Step 1: All the parameters to be optimized in the IBRB-HOAI model are encoded into a vector. This vector is referred to as a “particle”. Each particle is considered a candidate solution in the model’s parameter space. A particle swarm of size is subsequently randomly initialized $N_{\mathrm{pso}}$ within the search space that satisfies all the constraints.

(22) $$\Phi =\{\theta_{1},\theta_{2},\ldots ,\theta_{L},\beta_{1,1},\ldots ,\beta_{L,N},\delta_{1},\delta_{2},\ldots ,\delta_{L}\}$$

Step 2: In each iteration, the particle velocity v and position z are updated. Both updates are guided by the individual historical best position pbest and global best position gbest. The updates for the velocity and position are as follows:

(23) $$v_{i}(t+1)=w\cdot v_{i}(t)+q_{1}\cdot r_{1}\cdot (pbest_{i}-z_{i}(t))+q_{2}\cdot r_{2}\cdot (gbest-z_{i}(t))$$

(24) $$z_{i}(t+1)=z_{i}(t)+v_{i}(t+1)$$

where the inertia weight w is employed to balance global and local search; the learning factor is denoted by $q_{1},q_{2}$; and $r_{1},r_{2}$ are random numbers. After updating, boundary violations of the particle positions must be handled to ensure that all the model parameters consistently satisfy the physical constraints. The velocity of particle i is given by $v_{i}(t)$. The position of particle i is given by $z_{i}(t)$. The personal best position of a particle i is given by $pbest_{i}$. The global best position of a particle is given by gbest.

Boundary Constraint Handling:

(25) $$z_{i}(t+1)=\min\left(\max\left(z_{i}(t+1),lb_{i}\right),ub_{i}\right)$$

(26) $$v_{i}(t+1)=\min\left(\max\left(v_{i}(t+1),-v_{max}\right),v_{max}\right)$$

where the upper and lower bounds for the ith particle are defined as $ub_{i}$ and $lb_{i}$ respectively.

Step 3: Fitness calculation:

(27) $$f_{\mathrm{new}}=MSE(z_{i}(t+1))=\frac{1}{H}{\sum_{i=1}^{H}\left(y_{i}(z_{i}(t+1))-y_{i}^{actual}\right)}^{2}$$

where the fitness value of particle i at its new position $z_{i}(t+1)$ is $f_{\mathrm{new}}$.

Step 4: Update Individual Best:

(28) $$pbest_{i}=\left\{\begin{array}{ll} z_{i}(t+1) & \mathrm{if}\;f_{\mathrm{new}}<\mathrm{MSE}(pbest_{i})\\ pbest_{i} & \mathrm{otherwise} \end{array}\right.$$

Update Global Best:

(29) $$gbest=\arg\underset{pbest_{i}}{\min}\mathrm{MSE}(pbest_{i})$$

Step 5: Global optimal solution:

(30) $$z_{\mathrm{pso}}^{*}=gbest$$

where the optimal solution found by the PSO algorithm is $z_{\mathrm{pso}}^{*}$.

Final population:

(31) $$P_{\mathrm{pso}}=\left\{z_{1}(T_{\mathrm{pso}}),\ldots ,z_{N_{\mathrm{pso}}}(T_{\mathrm{pso}})\right\}$$

When the PSO iteration reaches the preset maximum generation $T_{pso}$, the first stage is concluded. At this point, the global best position gbest found via the PSO is regarded as a high-quality approximation of the global optimal solution. gbest and the surrounding search distribution information are taken as the initial inputs for the P-CMA-ES algorithm in the second stage. The output of the PSO is directly used as the starting point for the P-CMA-ES.

Upon completion of the PSO stage, the global best particle position $z_{\mathrm{pso}}^{*}$ is used as the initial mean $mean^{\left(0\right)}$ of the search distribution in the P-CMA-ES stage:

The parameters of the improved P-CMA-ES algorithm are initialized. The initial mean value is $mean^{\left(0\right)}=\Omega^{\left(0\right)}$. The parameter vector $\Omega^{\left(0\right)}$ obtained via PSO represents the global optimal solution. The initial covariance matrix is denoted as $C^{\left(0\right)}$. The initial step size is denoted as $\sigma^{\left(0\right)}$. The population size is λ. The offspring population size is set as τ. The value of $\sigma^{\left(0\right)}$ is calculated via the following formula:

Initial mean:

(32) $$\Omega^{\left(0\right)}\equiv z_{\mathrm{pso}}^{*}$$

Initial step size:

(33) $$\mu_{\mathrm{pso}}=\frac{1}{N_{\mathrm{pso}}}\sum_{i=1}^{N_{\mathrm{pso}}}x_{i}(T_{\mathrm{pso}})$$

(34) $$\sigma^{\left(0\right)}=\xi \cdot \sqrt{\frac{1}{N_{\mathrm{pso}}}\sum_{i=1}^{N_{\mathrm{pso}}}{\left\|x_{i}(T_{\mathrm{pso}})-\mu_{\mathrm{pso}}\right\|}^{2}}$$

Initial covariance matrix:

(35) $$C^{\left(0\right)}=\frac{1}{N_{\mathrm{pso}}-1}\sum_{i=1}^{N_{\mathrm{pso}}}\left(x_{i}(T_{\mathrm{pso}})-\mu_{\mathrm{pso}}\right){\left(x_{i}(T_{\mathrm{pso}})-\mu_{\mathrm{pso}}\right)}^{T}$$

Initialize the evolutionary path:

(36) $$P_{\sigma }^{\left(0\right)}=0,\;P_{c}^{\left(0\right)}=0$$

Step 6: Sampling operation. The initial population is generated as follows:

(37) $$\Omega_{k}^{\left(g+1\right)}=mean^{\left(g\right)}+\varepsilon^{\left(g\right)}N(0,C^{\left(g\right)}),k=1,2,\ldots ,\lambda$$

where $\Omega_{k}^{\left(g+1\right)}$ is the solution i of generation g+1. $mean^{\left(g\right)}$ is used to denote the mean of the search distribution for the sth generation. $C^{\left(g\right)}$ denotes the sth covariance matrix. The number of offspring is denoted by λ, while normal distribution is represented by N(•).

Step 7: Control operation. Through expert knowledge, erroneous confidence distributions are identified and resampled until all the distribution conditions are satisfied:

(38) $$\Omega_{k}^{\left(g+1\right)}⇐\beta_{k}^{\left(g+1\right)}=mean^{\left(g\right)}+\varepsilon^{\left(g\right)}N(0,C^{\left(g\right)})$$

where $\beta_{k}^{\left(g+1\right)}$ is the reasonable belief distribution, and ⇐ is the replacement operation that causes the incorrect belief distribution in $\Omega_{k}^{\left(g+1\right)}$ to be replaced.

Step 8: Projection Constraints:

(39) $$A_{e}\Omega_{k}^{\left(g+1\right)}(1+n_{e}\times (j-1):n_{e}\times j)=1,j=1,2,\ldots N+1$$

(40) $$\begin{array}{l} \Omega_{k}^{\left(g+1\right)}(1+n_{e}\times (j-1):n_{e}\times j)=\Omega_{k}^{\left(g+1\right)}(1+n_{e}\times (j-1):n_{e}\times j)\\ -A_{e}^{T}\times {\left(A_{e}{A_{e}}^{T}\right)}^{-1}\times \Omega_{k}^{\left(g+1\right)}(1+n_{e}\times (j-1):n_{e}\times j)\times A_{e} \end{array}$$

where $n_{e}=1,\ldots ,N$ denotes the variable with constraints, and j=1,…,N+1 indicates how many equation restrictions there are.

Step 9: Selection and Recombination:

(41) $$mean^{\left(g+1\right)}=\sum_{k=1}^{\tau }\psi_{k}\Omega_{k:\lambda }^{\left(g+1\right)}$$

where $\psi_{k}$ denotes the weight coefficient, and the size of the number of offspring is represented as τ. The symbol $\Omega_{k:\lambda }^{\left(g+1\right)}$ represents the ith solution of λ solutions of the g+1 generation’s search distribution.

Step 10: Update the evolution path (step size control):

(42) $$p_{\sigma }^{\left(g+1\right)}=(1-c_{\sigma })p_{\sigma }^{\left(g\right)}+\sqrt{c_{\sigma }(2-c_{\sigma })\mu_{\mathrm{eff}}}C^{{\left(g\right)}^{-\frac{1}{2}}}\frac{m^{\left(g+1\right)}-m^{\left(g\right)}}{\sigma^{\left(g\right)}}$$

where the effective population size is denoted by $\mu_{\mathrm{eff}}$, the time constant for cumulation for C is denoted by $c_{c}$, the time constant for cumulation for sigma control is denoted by $c_{\sigma }$, the learning rate for rank-one update of C is denoted by $c_{1}$, the learning rate for rank-mu update is denoted by $c_{\mu }$, the damping for sigma is denoted by $d_{\sigma }$. The respective formulas are as shown below.

(43) $$\mu_{\mathrm{eff}}=\frac{{\left(\sum_{i=1}^{\mu }w_{i}\right)}^{2}}{\sum_{i=1}^{\mu }w_{i}^{2}}$$

(44) $$c_{c}=\frac{4+\mu_{\mathrm{eff}}/N}{N+4+2\mu_{\mathrm{eff}}/N}$$

(45) $$c_{\sigma }=\frac{\mu_{\mathrm{eff}}+2}{N+\mu_{\mathrm{eff}}+5}$$

(46) $$c_{1}=\frac{2}{{\left(N+1.3\right)}^{2}+\mu_{\mathrm{eff}}}$$

(47) $$c_{\mu }=\min\left(1-c_{1},\frac{2(\mu_{\mathrm{eff}}-2+1/\mu_{\mathrm{eff}})}{{\left(N+2\right)}^{2}+\mu_{\mathrm{eff}}}\right)$$

(48) $$d_{\sigma }=1+2\max\left(0,\sqrt{\frac{\mu_{\mathrm{eff}}-1}{N+1}}-1\right)+c_{\sigma }$$

Update step size:

(49) $$\sigma^{\left(g+1\right)}=\sigma^{\left(g\right)}\exp\left(\frac{c_{\sigma }}{d_{\sigma }}\left(\frac{\left\|p_{\sigma }^{\left(g+1\right)}\right\|}{E\left[\left\|N(0,I)\right\|\right]}-1\right)\right)$$

Update the evolution path (covariance update):

(50) $$p_{c}^{\left(g+1\right)}=(1-c_{c})•p_{c}^{\left(g\right)}+\sqrt{c_{c}(2-c_{c})}•{\left(\sum_{i=1}^{\tau }\psi_{i}^{2}\right)}^{-0.5}•(mean^{\left(g+1\right)}-mean^{\left(g\right)})/\varepsilon^{\left(g\right)}$$

(51) $$h_{\sigma }^{\left(g+1\right)}=\left\{\begin{array}{ll} 1 & \mathrm{if}\;\frac{\left\|p_{\sigma }^{\left(g+1\right)}\right\|}{\sqrt{1-{\left(1-c_{\sigma }\right)}^{2(g+1)}}}<\chi_{D}\\ 0 & \mathrm{otherwise} \end{array}\right.$$

Update the covariance matrix:

(52) $$\begin{array}{l} C^{\left(g+1\right)}=(1-c_{1}-c_{2})•C^{\left(g\right)}+c_{1}p_{c}^{\left(g+1\right)}{\left(p_{c}^{\left(g+1\right)}\right)}^{T}\\ +c_{2}\sum_{k=1}^{\tau }\omega_{i}(\frac{\Omega_{k:\lambda }^{\left(g+1\right)}-mean^{\left(g\right)}}{\varepsilon^{\left(g\right)}}){\left(\frac{\Omega_{k:\lambda }^{\left(g+1\right)}-mean^{\left(g\right)}}{\varepsilon^{\left(g\right)}}\right)}^{T} \end{array}$$

where the generation step g is denoted as $\varepsilon^{\left(g\right)}$. The learning rates are denoted by $c_{1}$ and $c_{2}$. The generation g+1 evolutionary trajectory is represented by the letter $p_{c}^{\left(g+1\right)}$. $mean^{\left(g\right)}$ is a representation of the generation g of the offspring population.

**Figure 2:**
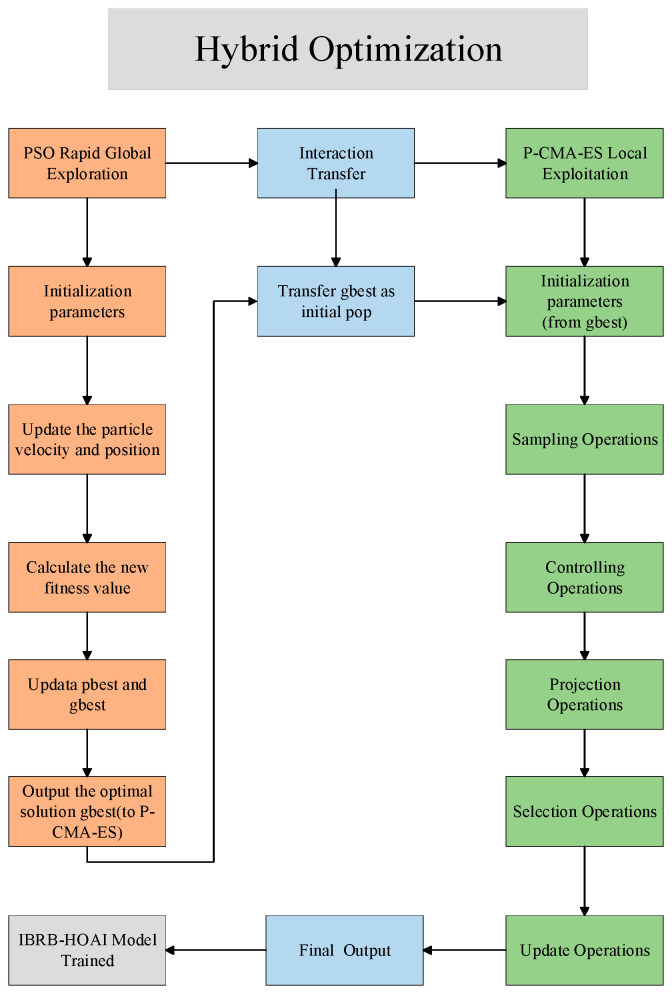
Hybrid Optimization Based on PSO and P-CMA-ES.

P-CMA-ES output:

(53) $$\Phi_{\mathrm{best}}=mean^{\left(G\right)}$$

(54) $$\Phi_{\mathrm{best}}\to (\theta^{*},\beta^{*},\delta^{*})$$

where G represents the maximum number of iterations. The iteration reaches the preset maximum generation, and the entire hybrid optimization process terminates. At this point, the final mean vector output by P-CMA-ES $\Phi_{\mathrm{best}}$ is the optimized parameter set of the IBRB-HOAI model. The optimization process is shown in Figure 2.

The PSO stage does not simply output a single point. Instead, the distribution characteristics of the solution space obtained through its search are passed to the P-CMA-ES, ensuring that the regional information acquired during the global search phase is not lost. Based on this initial distribution, local sampling and distribution updates are performed by the P-CMA-ES, thereby avoiding repeated search of already explored regions. In both stages, all optimized parameters are subjected to identical constraints and boundary conditions, ensuring the consistency and feasibility of the model structure throughout the interaction.

Through the above interaction mechanism, a smooth transition from “global coarse search” to “local fine search” is achieved by the hybrid optimization process, fully exploiting the complementary advantages of the two algorithms. All formula symbols are listed in Table A1 in Appendix A.

## 4. Case Study

A health assessment test is conducted on the WD615 diesel engine system. The effectiveness of the IBRB-HOAI model is verified through this test.

### 4.1. Experimental Definition

#### 4.1.1. Dataset Description

Diesel engines play a critical role in the automotive and transportation sectors. This is attributed to their high fuel utilization efficiency. Accurate HSA is considered increasingly important. This is due to rising demands for diesel engine performance and reliability. The data were collected through Sensor A1. A total of 2700 sets were obtained. The accuracy of the collected data is often compromised. This occurs because collected signals are easily affected by environmental noise during actual operation. The construction of a precise assessment model is considered challenging. This difficulty arises because diesel engine systems typically involve multiple interconnected sensors. Reliance on expert experience alone is often insufficient. The structural complexity is directly of the model influenced by the adopted reference intervals. As a result, the accuracy of HSA is significantly affected. Therefore, adaptive optimization of reference intervals is required during model training. This optimization should be aligned with actual operational conditions. The development of a more accurate method for diesel engine health assessment is essential. This is crucial for improving operational efficiency and extending service life.

#### 4.1.2. Experimental Settings

In the WD615 dataset [38], the steady-state speed is set at 1800 r/min. The sampling frequency is set at 12.8 kHz. Vibration sensors were installed at different positions on the engine. Piston-to-cylinder clearances were adjusted to three distinct values. These clearance adjustments are designed to simulate three engine operating states: normal operation (N), slight fault (S), and severe fault (SF). As shown in Table 4, cylinder vibration signals were subsequently collected from the diesel engine. These raw signals are difficult to analyze and compare directly. The instance validation in this study involved random sampling across three distinct health states of the diesel engine. A total of 2700 data samples were obtained. The numbers for the three health states are 2000, 200, and 500, respectively [39]. To ensure the fairness of data selection, these three health states are divided into training and testing sets. The split ratio is set at 7:3.

#### 4.1.3. Model Evaluation Metrics

Five evaluation metrics are introduced: accuracy, recall, precision, F1 score, and MSE. Descriptions of these metrics are provided below:

(1) Accuracy

Accuracy is defined as the proportion of correct predictions generated by the model. It is computed as the ratio of the number of correct predictions to the total number of predictions made.

(55) $$AC=\frac{NCP}{TNP}$$

(2) Precision

Precision is defined as a quantitative metric. It is calculated as the proportion of correctly detected positive cases among all cases predicted as positive.

(56) $$PC=\frac{TP}{TP+FP}$$

(3) Recall

Recall is defined as the proportion of true positive samples that are correctly identified by the model. It is also referred to as the true positive rate.

(57) $$RC=\frac{TP}{TP+FN}$$

(4) F1 score

The F1 score is defined as the harmonic mean of precision and recall. It serves as a comprehensive metric that balances both precision and recall. Its value ranges from 0 to 1. A higher F1 score indicates a better balance between accuracy and completeness, which reflects a stronger fault identification capability.

(58) $$F1=\frac{2\times PC\times RC}{PC+RC}$$

(5) MSE

The mean squared error (MSE) is defined as the average of the squared differences between the predicted values and the true values. It is a widely used metric for evaluating regression performance. Larger errors are penalized more heavily through the squaring term, which makes the metric more sensitive to outliers in predictions. MSE is a nonnegative scalar with values ranging from 0 to positive infinity. A lower MSE indicates a smaller overall deviation between the predicted and true values, reflecting higher prediction accuracy and stability.

(59) $$\mathrm{MSE}=\frac{1}{N}\sum_{i=1}^{N}{\left(x-x^{*}\right)}^{2}$$

#### 4.1.4. Evaluation Protocol and Performance Stability Guarantee

To ensure scientific rigor and reproducibility, this section specifies the data partitioning strategy and the measures taken to guarantee performance stability.

(1) Training/Testing Set Partitioning

All 2700 samples were divided into training and testing sets using a stratified random sampling method with a ratio of 7:3. The training set contains 1890 samples, and the testing set contains 810 samples. Stratification ensures that the class distributions of the three health states in both the training and testing sets are consistent with those of the original dataset, avoiding evaluation bias caused by class distribution shift. The training set is used for parameter learning, while the testing set is used only for final performance evaluation and is not exposed during model training or optimization.

(2) Validation Strategy

During model training, a 5-fold cross-validation strategy is applied within the training set. Specifically, the training set is randomly partitioned into five mutually exclusive and approximately equal-sized subsets. In each fold, four subsets are used for training and the remaining one for validation. This process is repeated five times until each subset has served as the validation set once. The optimal hyperparameters are selected based on the average performance across the five folds. This strategy makes efficient use of the limited training data while reducing the randomness introduced by a single validation split. The results of the 5-fold cross-validation described in this section are shown in the comparative experiments of this chapter.

(3) Performance Stability Assurance

To ensure the statistical reliability of the evaluation results and the generalization stability of the model, the following measures were taken. To eliminate the influence of random data partitioning, the complete experimental procedure described above was independently repeated 20 times. The final reported MSE and accuracy metrics are shown in the comparative experiments of this chapter., respectively, thereby quantifying the performance fluctuation range of the model under different data samplings. For comparison with baseline methods, the Wilcoxon signed-rank test was applied to statistically analyze the MSE results from the 20 repeated experiments, and the Bonferroni correction was used to control the error rate of multiple comparisons. A statistically significant performance improvement of the proposed method was determined when the corrected *p*-value was less than 0.05.

Through the measures described above, the reported performance metrics exhibit good reproducibility and statistical reliability, thus objectively reflecting the generalization ability of the model under different data partitions.

In summary, the scientific validity, reproducibility, and statistical reliability of the experimental evaluation were ensured through a clear stratified 7:3 training/testing set split and a 5-fold cross-validation strategy, combined with stability assurance measures such as 20 repeated experiments and statistical significance testing.

### 4.2. Initial Model Parameter Configuration

In engine health assessments based on vibration signals, effectively selecting the most contributive parameters from high-dimensional and nonlinear feature sets is key to constructing a reliable diagnostic model. The random forest method is adopted for feature selection. This approach is justified by its deep alignment with vibration signal analysis in both theoretical mechanisms and application scenarios. The rationale is specifically reflected in the following aspects.

First, regarding the alignment between signal characteristics and model mechanisms, engine vibration signals often exhibit non-stationary, non-Gaussian, and nonlinearly coupled characteristics. As an ensemble learning method, the core mechanism of random forest involves constructing multiple decision trees and performing random subspace sampling on features. This enables the natural capture of nonlinear interactions and high-order dependencies among features, without relying on linear or parametric assumptions. This characteristic makes it particularly suitable for handling the complex mapping relationships between vibration signals and health states in practical engineering applications.

Second, by calculating the impurity reduction contributed by each feature during the splitting processes across numerous decision trees, a stable importance score can be assigned to every feature. Compared with filter methods that rely on statistical correlation, this evaluation method, which is based on internal model performance contributions, more accurately reflects the actual utility of features in classification. Furthermore, it provides a clear quantitative basis for subsequent feature screening.

Furthermore, from the perspective of engineering practicality and generalization capability, engine monitoring data often face challenges such as limited sample size, noise interference, and feature redundancy. Through bootstrap sampling and random feature selection, the random forest method effectively reduces the model’s sensitivity to noise and overfitting, enhancing its stability in high-dimensional, small-sample scenarios. This ensures that the selected feature subset is not only effective on the current dataset but also has potential for transfer to similar vibration diagnosis tasks.

**Figure 3:**
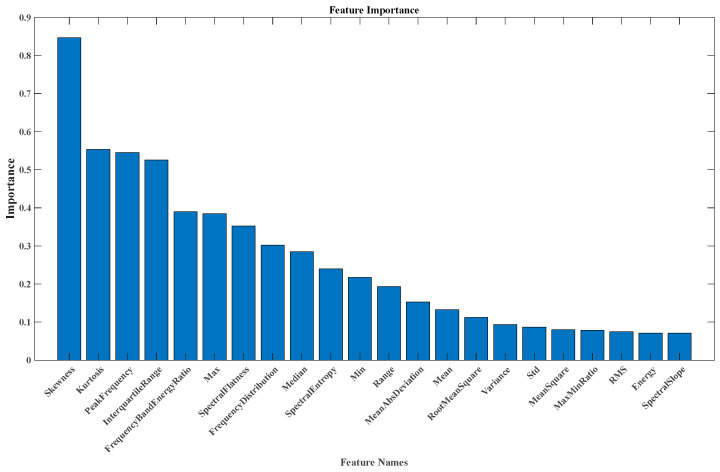
Feature Importance Ranking Based on the Random Forest Method.

In this study, the random forest algorithm is employed to rank the importance of 22 attributes, so as to select the core features that contribute most to the health assessment model. The results of feature importance ranking are shown in Figure 3. The scores reflect the average contribution of each feature to reducing the prediction error of the model. To avoid doubts caused by subjective truncation, the rationality of selecting the top four features (Skewness, Kurtosis, PeakFrequency, InterquartileRange) is systematically demonstrated below. From the feature importance distribution curve, a significant stepwise decreasing trend is observed. The importance values are as follows: Skewness (≈0.85), Kurtosis (≈0.55), PeakFrequency (≈0.54), and InterquartileRange (≈0.52). The importance scores of these four features are significantly higher than those of the subsequent features, forming a clear “plateau period”. This distribution conforms to the Elbow method in statistics.

In the feature importance curve, features before the “elbow”, i.e., the inflection point where scores decrease sharply, are statistically significant in their contribution to the model output. The first four features are selected because they form the “elbow” of the importance distribution. They represent the core information dimensions that contribute most to the prediction ability of the model. This truncation method avoids subjective arbitrariness and provides an objective statistical basis for feature selection. In machine learning modeling, a larger number of features is not always better. Excessive irrelevant or weakly correlated features increase model complexity and cause overfitting. Meanwhile, the interpretability of the model is reduced. The advantages of selecting the top four features are as follows. The feature space is compressed from 22 dimensions to 4 dimensions. The computational burden and overfitting risk of the model are significantly reduced. Good generalization ability is maintained even with limited training data. A small number of core features make the decision-making process of the model more transparent. The prediction results of the model can be easily explained from a mechanistic perspective. Therefore, the random forest method is used to rank the extracted features based on their importance, from which key features are selected.

In the preliminary feature importance analysis, the random forest algorithm was employed to rank the importance of the original feature set. The results indicated that four features—skewness, kurtosis, peak frequency, and interquartile range—formed a distinct “elbow point” in the importance curve, after which the importance of subsequent features decreased significantly. To enhance the methodological rigor of the feature selection process and avoid reliance solely on subjective visual judgment, an ablation study was further designed to systematically evaluate the impact of different feature quantities on model performance and computational efficiency.

Specifically, based on the importance ranking derived from the random forest, three feature subsets were constructed, containing the top 3, 4, and 5 most important features, respectively. For each subset, models were trained under identical experimental settings, and the following metrics were recorded: (1) mean squared error (MSE) and accuracy (AC) were adopted as the primary evaluation metrics. The mean values were calculated over 20 experimental runs to assess the predictive ability of the models under different feature quantities. (2) The average time consumption (in seconds) during the training and inference phases was recorded to quantify the impact of feature dimensionality on computational efficiency.

The experimental results are summarized in Table 5. The results revealed that when the number of features increased from 3 to 4, the prediction performance improved significantly (the MSE decreased by approximately 7.4%), whereas computational cost increased only slightly (by approximately 15.8%). When the number of features was further increased to 5, the improvement in predictive performance slowed (the MSE decreased by approximately 5.3%) and computational cost continued to increase (by approximately 14%). By balancing predictive performance and the computational efficiency, retaining the top four features (skewness, kurtosis, peak frequency, and interquartile range) maintained high prediction accuracy while keeping computational overhead low, thereby validating the rationality of the feature selection strategy.

Through the ablation study described above, a quantitative basis was provided for feature quantity selection. The methodological limitations of relying solely on subjective visual identification of the elbow point were addressed, and the reproducibility and persuasiveness of the feature selection process were enhanced.

The figure clearly shows that higher feature importance is more beneficial for model construction. Therefore, in this experiment, the following four features were selected: skewness, kurtosis, peak frequency, and interquartile range. These attributes were chosen as essential inputs for the IBRB-HOAI model. In this experiment, seven reference intervals were defined for each feature: very low (VL), somewhat low (SL), low (L), medium (M), somewhat high (SH), high (H), and very high (VH). The corresponding reference values were provided by experts on the basis of engine characteristics, as shown in Table 6. The accuracy and complexity of the health assessment model are influenced by the number of reference points. Table 7 presents the initial belief table. Thus, the kth rule in the initial IBRB-HOAI can be expressed as follows:

(60) $$\begin{array}{l} R_{k}:If(Skewness\;is\;RI_{1})\vee (\mathrm{Kurtosis}\;is\;RI_{2})\vee \\ \left(PeakFrequency\;is\;RI_{3})\vee (InterquartileRange\;is\;RI_{4}\right)\\ \;Then\;health\;states\;is\{(N,\beta_{1,k}),(S,\beta_{2,k}),(SF,\beta_{N,k})\}\\ \;with\;rule\;weight\;\theta_{k}\;\mathrm{and}\;rule\;reliability\;\delta_{k}\;k\in \{1,2,3\}\\ \;\sum_{i=1}^{3}\beta_{i,k}\leq 1\; \end{array}$$

**Figure 4:**
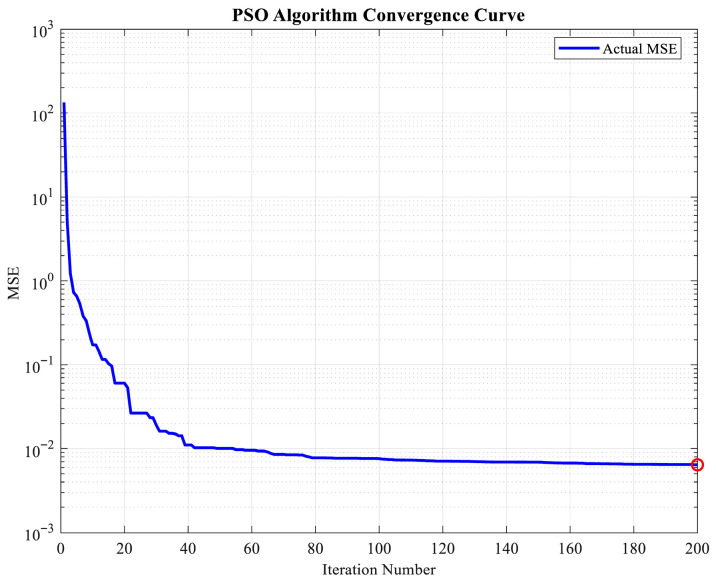
Convergence Analysis of PSO.

The convergence curve of the PSO algorithm, it revealed that the mean squared error (MSE) decreased rapidly as the number of iterations increased. In the first 50 iterations, a sharp decline in the MSE was observed. This finding indicates that a relatively optimal solution space was quickly searched by the algorithm in the initial stage. Between 50 and 150 iterations, the MSE was further reduced slowly to approximately $10^{-2}$. During this phase, the algorithm entered a fine search stage, gradually approaching the global optimal solution. When the number of iterations reached 200, the MSE stabilized on the order of $10^{-3}$. In subsequent iterations, no significant further reduction in error was observed, and the curve became flat. This finding indicates that convergence was achieved by the algorithm. The selection of 200 iterations as the termination condition was based on the following reasons: First, at 200 iterations, the MSE had converged to a stable value. Further increasing the number of iterations would have provided limited improvement in solution quality while significantly increasing the computational cost. Second, from an engineering application perspective, a reasonable balance between computational efficiency and optimization accuracy was achieved with 200 iterations, provided that solution quality was ensured. Additionally, a stable trend was shown in the convergence curve after 200 iterations, further confirming that the convergence criterion was satisfied by the algorithm under this iteration count. Therefore, the maximum number of iterations for the PSO algorithm was set to 200 in this study. In this way, both the reliability of the optimization results and the computational efficiency were considered. The results are presented in Table 6. The convergence analysis of the PSO is shown in Figure 4.

### 4.3. Experimental Analysis

The experimental results in this section are analyzed. The analysis includes comparisons between the IBRB-HOAI model and traditional IBRB models with fixed reference intervals, as well as comparisons with IBRB models using only constrained PSO or only P-CMA-ES for optimization. Table 8 presents the optimized parameters obtained through the IBRB-HOAI model, while Table 9 displays the adaptive reference intervals.

**Figure 5:**
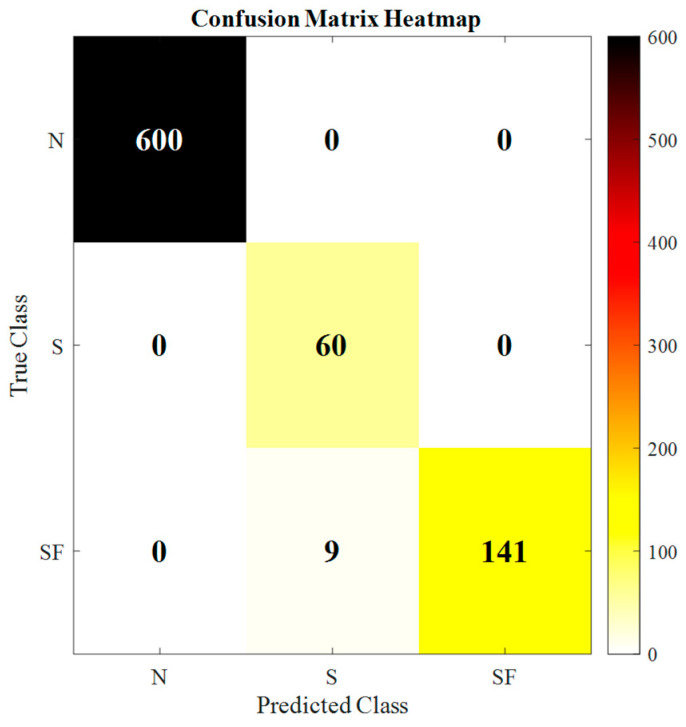
Confusion matrix for the IBRB-HOAI.

The adaptive reference interval optimization mechanism introduced in the IBRB model is key for improving model performance. By dynamically adjusting the reference intervals of the belief rule antecedents, the model structure is freed from the constraints of traditional fixed intervals. This enables the model to align more accurately with the actual distribution of the data. This approach significantly enhances the expressive power and discriminative ability of belief rules. It also has a synergistic effect with the subsequent hybrid optimization algorithm, jointly guiding the model toward a globally superior solution. Theoretical analysis indicates that this design forms the foundation for improving both the fitting ability and the generalization performance of the model. Its effectiveness will be validated in subsequent comparative experiments. As shown in the confusion matrix in Figure 5, the IBRB-HOAI model achieves an accuracy of 98.27%.

The IBRB-HOAI model successfully achieves precise health assessment for diesel engines, accurately distinguishing between normal operation, slight faults, and severe faults. Its near-perfect overall performance, particularly its absolute discriminative ability between normal and severe fault states, validates the effectiveness and significant potential of applying adaptive reference interval optimization combined with a hybrid algorithm to the IBRB model for addressing complex system health assessment problems. This model provides a robust theoretical tool for achieving highly reliable predictive maintenance of equipment.

**Figure 6:**
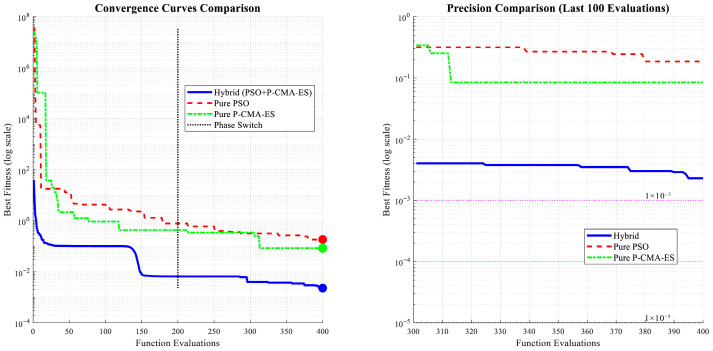
Convergence speed and precision of the hybrid optimization algorithm versus the stand-alone algorithms.

To comprehensively evaluate the performance of the proposed hybrid optimization algorithm (PSO + P-CMA-ES), this study conducts a comparative analysis against pure PSO, a pure P-CMA-ES, and a phased switching strategy (Phase Switch). The comparison focuses on three dimensions: convergence speed, convergence precision, and stability. All the experiments were conducted under identical initial conditions and with the same maximum number of evaluations (400 function evaluations). The results are shown in Figure 6 below.

**Figure 7:**
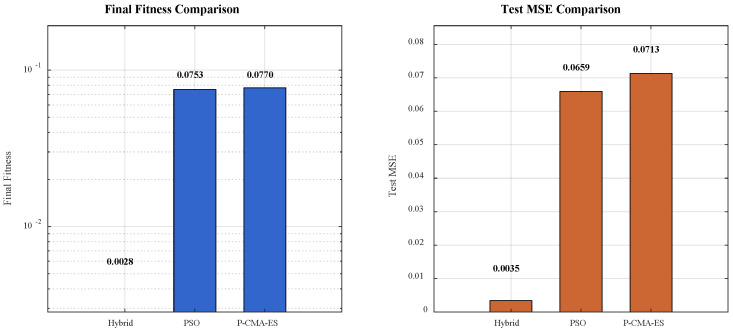
Experimental analysis.

The hybrid algorithm (PSO + P-CMA-ES) demonstrates the optimal convergence characteristics. Its curve maintains a rapid declining trend throughout the evaluation process and stabilizes after approximately 150 evaluations, exhibiting the dual advantages of fast convergence and early stabilization. The pure PSO algorithm converges rapidly in the early stage but falls into local optima later. Within the first 100 evaluations, PSO has the fastest convergence speed. However, its progress slows significantly afterward, and the curve flattens, indicating insufficient global exploration capability. The pure P-CMA-ES algorithm converges relatively slowly in the early stage but demonstrates sustained optimization in the mid-to-late stages. Initially, it lags behind PSO. However, after approximately 200 evaluations, it gradually approaches and eventually surpasses PSO, indicating strong local exploitation capability. The phased switching strategy shows performance between the two. Its curve reflects a change in convergence behavior after the switching point (approximately 150 evaluations). However, its overall performance does not surpass that of the hybrid algorithm. These results further validate the effectiveness of the hybrid strategy. The global exploration capability of PSO and the local exploitation capability of P-CMA-ES complement each other within the hybrid framework. This enables the algorithm to continue approaching the global optimum even in the late convergence stage. To verify the generalization performance and convergence quality of the proposed hybrid optimization algorithm (PSO + P-CMA-ES) on practical problems, this study further compared the final fitness values and tested the mean squared error (MSE) of each algorithm on an independent test set. The results indicate that the hybrid algorithm significantly outperforms both pure PSO and the pure P-CMA-ES in terms of both optimization objectives and predictive performance. The results are shown in Figure 7.

**Figure 8:**
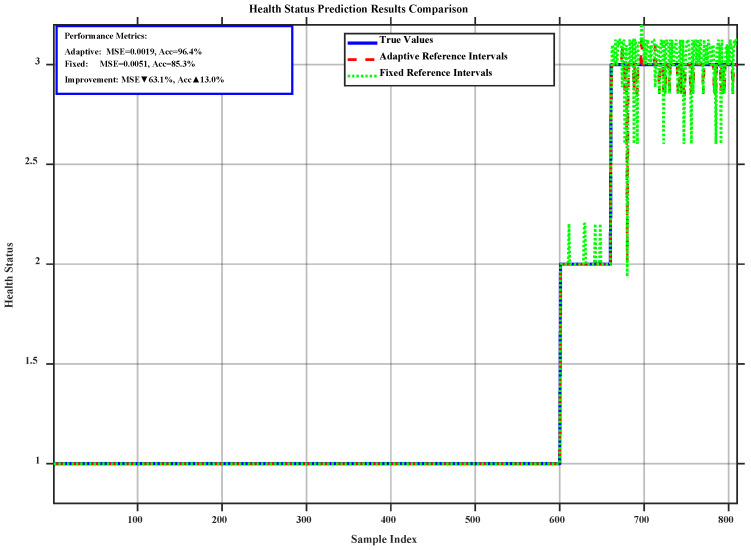
Comparison of MSE convergence curves between the adaptive reference interval and the fixed reference interval in a single typical.

To validate the effectiveness of the proposed adaptive reference interval adjustment mechanism in practical applications, this study compares it with the fixed reference interval method using the same dataset. The experiment involved 810 samples and was evaluated via two core metrics: the MSE and the classification accuracy. The results are shown in Figure 8. Compared with the fixed mechanism, the adaptive mechanism reduces the MSE by approximately 63.1%. This indicates a significant decrease in the deviation between the predicted and actual values, indicating higher prediction precision. Furthermore, the adaptive mechanism achieves an accuracy of 96.4%. This represents an improvement of approximately 13.0% over the fixed mechanism, confirming its superior classification performance in health state discrimination.

### 4.4. Comparative Experiments

To analyze the comprehensive performance of the IBRB-HOAI model, a comparative study was conducted against a backpropagation neural network (BPNN), K-nearest neighbors (KNN), decision tree (DT), extreme learning machine (ELM), logistic regression (LR), support vector machine (SVM) and convolutional neural network (CNN). Comparisons were carried out across five core classification metrics: AC, PC, RC, F1 score, and MSE. The specific results are presented in Table 10.

The experimental results indicate that the IBRB-HOAI model is a gray-box model capable of integrating observational data with expert knowledge for assessment. A comparative study was conducted with black-box modeling methods such as BPNN, KNN, decision tree, ELM, logistic regression, SVM, and CNN. The proposed IBRB-HOAI model significantly outperforms all the compared models across all the evaluation metrics. Specifically, the IBRB-HOAI achieves an accuracy of 97.52%, representing an improvement of approximately 2.81 percentage points over the next best-performing model, CNN (94.71%). Compared with other traditional models, its advantage is even more pronounced, with improvements ranging from 3.12% to 6.01%. In terms of precision, the IBRB-HOAI achieves 97.31%, which is approximately 3.33 percentage points higher than that of the second-ranked CNN model (93.98%), and 4.40 percentage points higher than that of the ELM model (92.91%). This further demonstrates its superior ability to reduce false positives. In terms of recall, the IBRB-HOAI achieves 97.15%, which is approximately 3.14 percentage points higher than that of the CNN model (94.01%). For the F1 score, the IBRB-HOAI achieves 97.25%, representing an improvement of approximately 2.74 percentage points over the CNN model (94.51%). This indicates clear advantages in both the completeness of identifying positive samples and the overall balance of classification performance. In terms of mean square error, the IBRB-HOAI achieves an MSE of 0.002176, which is substantially lower than those of the other compared methods. In comparison, BPNN achieves 0.00503, CNN achieves 0.0052123, ELM achieves 0.089708, KNN achieves 0.04575, decision tree achieves 0.04688, and logistic regression achieves 0.042914. The MSE of the IBRB-HOAI is reduced by approximately 56.7% compared with the second-best method, the BPNN, and by approximately 97.6% compared with the ELM, further validating its superior prediction accuracy. Overall, the IBRB-HOAI not only leads comprehensively across all the metrics, with an overall performance close to 97.5%, but also significantly surpasses various advanced models, including the CNN and the ELM. This fully validates the model’s effectiveness and robustness in handling complex classification tasks, demonstrating strong theoretical advantages and practical application value.

In addition, to comprehensively evaluate the effectiveness of the proposed IBRB-HOAI model, it was compared against multiple optimization models based on the same IBRB framework. These include the IBRB-PCMA model optimized via the P-CMA-ES algorithm, the IBRB-PSO model optimized via the PSO algorithm, the IBRB-GA model optimized via the genetic algorithm, and the IBRB-DE model optimized via the differential evolution algorithm. The experimental results are presented in the table. In terms of accuracy, the IBRB-HOAI achieves 97.52%, significantly outperforming the IBRB-PCMA (91.49%), IBRB-PSO (90.80%), IBRB-GA (90.51%), and IBRB-DE (90.62%), with improvements of approximately 6.03%, 6.72%, 7.01%, and 6.90%, respectively. In terms of precision, the IBRB-HOAI achieves 97.31%, which is approximately 6.50 percentage points higher than the IBRB-PCMA (90.81%) and 7.07 percentage points higher than IBRB-PSO (90.24%). In terms of recall, the IBRB-HOAI achieves 97.15%, representing an improvement of approximately 6.62 percentage points over IBRB-PCMA (90.53%). For the F1 score, the IBRB-HOAI achieves 97.25%, which is approximately 6.61 percentage points higher than IBRB-PCMA (90.64%). In terms of the mean square error, the IBRB-HOAI achieves an MSE of 0.002176, which is substantially lower than those of IBRB-PCMA (0.003771), IBRB-PSO (0.004223), IBRB-GA (0.004651), and IBRB-DE (0.004312), representing reductions of approximately 42.3%, 48.5%, 53.2%, and 49.5%, respectively. In summary, the proposed IBRB-HOAI model significantly outperforms other optimization models based on the IBRB framework across all evaluation metrics. This fully validates the superiority of the proposed hybrid optimization strategy (PSO for global exploration and P-CMA-ES for local refinement) over single optimization algorithms (P-CMA-ES, PSO, GA, DE), demonstrating the significant advantages of the IBRB-HOAI model in terms of prediction accuracy and classification performance. The results are presented in Table 11.

As shown by the 20-round experimental results in Table 12 and Table 13, the proposed IBRB-HOAI model demonstrates significant advantages in terms of both the MSE and AC across different training proportions (90%, 80%, 70%, 50%, and 30%). Overall, the IBRB-HOAI achieves the lowest MSE values across all training proportions, significantly outperforming both IBRB-PCMA and IBRB-PSO. Specifically, at a training proportion of 70%, the IBRB-HOAI attains its minimum MSE of 0.002176, which is approximately 42.3% lower than that of IBRB-PCMA (0.003771) and 48.5% lower than that of IBRB-PSO (0.004223). At training proportions of 50% and 30%, the MSE values of IBRB-HOAI are 0.002663 and 0.002735, respectively. Although these values increase slightly, they remain substantially lower than those of the other two models. Notably, the MSE values of the IBRB-PCMA and IBRB-PSO clearly increase as the training proportion decreases. When the training proportion is reduced from 90% to 30%, the MSE of IBRB-PCMA increases from 0.003245 to 0.004217, representing an increase of approximately 30.0%, while the MSE of the IBRB-PSO increases from 0.003579 to 0.004832, representing an increase of approximately 35.0%. In contrast, the MSE of IBRB-HOAI fluctuates only slightly with changes in the training proportion, demonstrating stronger stability and robustness. In summary, the IBRB-HOAI maintains optimal prediction accuracy across different training data proportions, consistently achieving the lowest MSE values and being least affected by the reduction in training data. This fully validates the significant advantages of the proposed hybrid optimization strategy in enhancing model generalization capability and stability.

In terms of time complexity, the average running time of the IBRB-HOAI method proposed in this paper (based on 20 experiments) was 32.41 s. The running times of the IBRB-PSO and IBRB-PCMAES methods were 34.48 s and 33.49 s, respectively. The experimental results that the IBRB-HOAI outperforms the two comparison methods in terms of computational efficiency. Compared with IBRB-PSO and IBRB-PCMAES, the running time was reduced by approximately 6.0% and 3.2%, respectively. This improvement is attributed mainly to the faster convergence speed achieved by the HOAI algorithm during parameter optimization. As a result, the computational cost of model training was effectively reduced. The results are presented in Table 14.

To evaluate the stability and generalization ability of the proposed model, a five-fold cross-validation method was employed. The dataset was divided into five mutually exclusive subsets. In each fold, four subsets were selected as the training set, and the remaining subset was used as the validation set. This process was repeated five times, and five sets of evaluation metrics were obtained. The results are shown in Table 15. Under five-fold cross-validation, the mean squared error (MSE) values were 0.003639, 0.001417, 0.000798, 0.001332, and 0.001664, respectively. The corresponding accuracy (AC) values were 97.07%, 95.85%, 95.94%, 96.24%, and 97.81%, respectively. After calculation, the average MSE was 0.00177, and the average accuracy was 96.58%. Small fluctuations were observed across the five folds. These findings indicate that the proposed model possesses good stability and generalization ability. High evaluation performance was consistently maintained under different data partitions.

Through K-fold cross-validation, the model maintained high accuracy and low error on unseen validation sets across all folds. No significant performance degradation was observed. These findings indicate that the model possesses strong generalizability, with no clear signs of overfitting.

To verify the effectiveness of the proposed IBRB-HOAI method and the statistical significance of its performance gains, an ablation study was conducted with four comparative methods. All the experiments were conducted on the same diesel engine dataset, which comprises 2700 sample points. To mitigate the impact of randomness on the experimental results, each method was independently repeated 20 times, and the mean square error (MSE) was recorded as the performance evaluation metric. Table 16 summarizes the MSE results of each method over 20 independent repeated experiments. To validate the statistical significance of the performance improvement achieved by the proposed IBRB-HOAI method, the Wilcoxon signed-rank test was employed to statistically analyze the MSE results from the 20 independent repeated experiments.

The results of the Wilcoxon signed-rank test indicate that compared with the IBRB, IBRB-PSO, and IBRB-PCMAES methods, the proposed IBRB-HOAI method results in statistically significant differences in terms of the MSE, with all the Bonferroni-corrected p-values less than 0.001. These findings strongly validate the superiority of the IBRB-HOAI method in terms of prediction accuracy. The results are presented in Table 17.

### 4.5. Experimental Summary

To comprehensively evaluate the proposed IBRB-HOAI model, two sets of comparative experiments were designed and conducted. The first set of experiments positioned the model within a broader algorithmic landscape, comparing it horizontally against various classical and advanced machine learning models, including BPNN, KNN, decision tree, ELM, and logistic regression. The second set of experiments aimed to verify the effectiveness of the hybrid optimization algorithm compared with single optimization algorithms. In this set, the IBRB-HOAI was compared against models optimized via the IBRB-PCMA and the standard IBRB-PSO.

The experimental results demonstrate that the proposed IBRB-HOAI model has significant advantages across all the performance metrics. First, in the horizontal comparison with various mainstream machine learning models, the IBRB-HOAI consistently shows superior performance. It leads comprehensively across the five metrics: AC, PC, RC, F1 score, and MSE. This finding not only confirms that the IBRB model possesses a robust theoretical framework for handling complex uncertainty problems but also highlights that the IBRB-HOAI model, optimized by the proposed hybrid strategy, outperforms a series of classical and cutting-edge machine learning models in terms of comprehensive performance. It achieves higher classification accuracy, stronger generalization capability, and superior overall performance. Second, within the internal comparison of the IBRB model family, IBRB-HOAI significantly outperforms the versions optimized solely with P-CMA-ES or PSO. These results strongly validate that the proposed IBRB-HOAI achieves a better balance between global exploration and local exploitation. Its optimization efficiency and stability are superior to those of single optimization algorithms, effectively preventing the model from becoming trapped in local optima.

In summary, the series of comparative experiments validates the effectiveness and advancement of the proposed model from multiple dimensions. Compared with various variants of IBRBs or competing with multiple mainstream machine learning algorithms, the IBRB-HOAI has outstanding performance. This confirms that the strategy of combining adaptive reference interval optimization with the hybrid P-CMA-ES and PSO algorithm is both successful and essential. It provides a superior solution for addressing this category of problem.

## 5. Conclusions

This research addresses the key scientific problem of diesel engine health state assessment. This model is based on a hybrid optimization strategy and adaptive reference intervals. The IBRB serves as the core reasoning engine of the method, effectively handling imprecision in quantitative data, uncertainty in qualitative knowledge, and issues related to missing information. The model dynamically tracks normal fluctuations in engine parameters through the introduction of an adaptive reference interval mechanism, providing a personalized health benchmark for different operational phases and overcoming the limitations of fixed-threshold methods in terms of adaptability. In addition, to address the challenge of model parameter optimization, a hybrid optimization strategy is designed. It effectively integrates global exploration with local optimization capabilities, significantly enhancing training efficiency and precision. As a result, the reliability and accuracy of the evaluation model are ensured.

Though the proposed method demonstrates satisfactory performance in diesel engine health assessment, its application still has certain limitations. First, the model construction relies on a certain amount of historical operational data to establish accurate reference intervals and rule bases. Its performance may be limited in scenarios with scarce data or incomplete coverage of operating conditions. Furthermore, although the hybrid optimization strategy improves parameter training effectiveness, its relatively high computational complexity may necessitate further efficiency optimization in online evaluation scenarios with stringent real-time requirements.

Future research could explore the following directions: First, by using health assessment results as inputs, a remaining useful life prediction model can be constructed. This would achieve a closed loop from condition assessment to life prediction, thereby providing more proactive support for maintenance decision-making. Second, the integration of multi-source information could be considered to further enrich the assessment dimensions and enhance the system’s robustness and comprehensive judgment capability. In addition, systematic analyses of hyperparameter sensitivity, noise robustness, and algorithm stability should be strengthened in future work. Further validation of the method’s generalization ability and engineering applicability should also be conducted through testing on multiple datasets in industrial scenarios.

## Figures and Tables

**Table 1:** BRB belief table.

Number	Point 1	Output
1	$\left[a_{1},b_{1}\right]$	{(N,0.9),(S,0.1),(SF,0)}
2	$\left[a_{2},b_{2}\right]$	{(N,0.8),(S,0.2),(SF,0)}
3	$\left[a_{3},b_{3}\right]$	{(N,0.7),(S,0.2),(SF,0.1)}
4	$\left[a_{4},b_{4}\right]$	{(N,0.5),(S,0.3),(SF,0.2)}
5	$\left[c_{1},d_{1}\right]$	{(N,0.3),(S,0.3),(SF,0.4)}
6	$\left[c_{2},d_{2}\right]$	{(N,0.2),(S,0.3),(SF,0.5)}
7	$\left[c_{3},d_{3}\right]$	{(N,0.1),(S,0.3),(SF,0.6)}
8	$\left[c_{4},d_{4}\right]$	{(N,0.0),(S,0.2),(SF,0.8)}

**Table 2:** Hyperparameter settings for the optimization algorithm.

Algorithm	Parameter Name	Symbol	Value	Description
PSO	Swarm size	$N_{\mathrm{pso}}$	40	The number of particles
	Maximum iterations (PSO phase)	$T_{\mathrm{pso}}$	200	The Global exploration stage
	Inertia weight	w	0.729	The balances global and local search
	Cognitive coefficient	$q_{1}$	1.49445	The individual experience factor
	Social coefficient	$q_{2}$	1.49445	The social interaction factor
	Velocity bound	$v_{\max}$	0.2×(ub−lb)	Prevents particle explosion
P-CMA-ES	Population size (offspring number)	λ	10+3lnN	
	Parent number	μ	$\left\lfloor \frac{\lambda }{2}\right\rfloor$	The number of parents for recombination
	Maximum evaluations (P-CMA-ES phase)	G	200	The local refinement stage
	Initial step size	$\sigma^{\left(0\right)}$	0.5	The coordinate-wise standard deviation
	Initial covariance matrix	$C^{\left(0\right)}$	-	The identity matrix
Hybrid	Total evaluations	G−total	400	The sum of two phases
	PSO→P-CMA-ES initialization	$mean^{\left(0\right)}$	-	The P-CMA-ES mean set to PSO optimum
	Switching condition	-	-	The fixed serial mechanism

**Table 3:** Switching Rule Configuration.

Mechanism Element	Description
Switching Condition	After PSO reaches the preset maximum number of iterations ($T_{\mathrm{pso}}$= 200), the switch is triggered unconditionally
Switching Action	The global optimal solution $z_{\mathrm{pso}}^{*}$ from PSO is used as the initial mean $mean^{\left(0\right)}$ for P-CMA-ES
Fallback Mechanism	None. Once P-CMA-ES completes, it does not return to PSO
Termination Condition	The total number of evaluations reaches G−total = 400

**Table 4:** Three Health States.

Health States	N	S	SF
Reference Value	1	2	3

**Table 5:** Ablation study results for feature selection.

Number	MSE	AC	Time
Three	0.002351	97.01%	27.97501
Four	0.002176	97.52%	32.40634
Five	0.002291	97.38%	39.92837

**Table 6:** Initial reference intervals for attributes.

Attribute	Referential Intervals						
Level	VL	SL	L	M	SH	H	VH
Skewness	[0.039, 0.4]	[0.4, 0.8]	[0.8, 1.2]	[1.2, 1.6]	[1.6, 2]	[2, 2.5]	[2.5, 2.91]
Kurtosis	[10, 30]	[30, 50]	[50, 70]	[70, 90]	[90, 110]	[110, 125]	[125, 140]
PeakFrequency	[37, 70]	[70, 2000]	[2000, 2300]	[2300, 2600]	[2600, 2900]	[2900, 3200]	[3200, 3665]
InterquartileRange	[0.12, 0.128]	[0.128, 0.136]	[0.136, 0.144]	[0.144, 0.152]	[0.152, 0.16]	[0.16, 0.17]	[0.17, 0.18]

**Table 7:** Initial belief table.

NO.	N	S	SF	NO.	N	S	SF	NO.	N	S	SF	NO.	N	S	SF
1	0.92	0.05	0.03	8	0.80	0.15	0.05	15	0.65	0.25	0.10	22	0.45	0.35	0.20
2	0.88	0.08	0.04	9	0.75	0.18	0.07	16	0.60	0.27	0.13	23	0.40	0.35	0.25
3	0.85	0.10	0.05	10	0.70	0.20	0.10	17	0.55	0.29	0.16	24	0.35	0.35	0.30
4	0.82	0.12	0.06	11	0.65	0.22	0.13	18	0.50	0.30	0.20	25	0.30	0.35	0.35
5	0.78	0.15	0.07	12	0.60	0.25	0.15	19	0.45	0.31	0.24	26	0.25	0.34	0.41
6	0.75	0.18	0.07	13	0.55	0.28	0.17	20	0.40	0.32	0.28	27	0.20	0.33	0.47
7	0.72	0.20	0.08	14	0.50	0.30	0.20	21	0.35	0.33	0.32	28	0.15	0.32	0.53

**Table 8:** Optimized parameter table.

Rule	Rule Reliability	Rule Reliability	N	S	SF
1	0.310932	0.399919	0.416981	0.39418	0.188839
2	0.525774	0.564737	0.17561	0.612217	0.212173
3	0.144227	0.093733	0.160847	0.729681	0.109472
4	0.044071	0.405655	0.619426	0.237084	0.14349
5	0.676526	0.42521	0.337111	0.552907	0.109982
6	0.865006	0.175792	0.220596	0.164842	0.614562
7	0.696939	0.765578	0.171683	0.762424	0.065893
8	0.09964	0.505035	0.058876	0.183562	0.757561
9	0.105625	0.455707	0.256879	0.055036	0.688085
10	0.450532	0.271709	0.38177	0.298512	0.319717
11	0.602647	0.474839	0.249261	0.685158	0.065581
12	0.391152	0.571641	0.451437	0.302952	0.245611
13	0.543619	0.631504	0.495087	0.450246	0.054667
14	0.558386	0.870646	0.249107	0.347874	0.40302
15	0.340624	0.572422	0.317241	0.185131	0.497628
16	0.596895	0.225896	0.48555	0.384325	0.130125
17	0.918442	0.568306	0.051009	0.221542	0.727449
18	0.813116	0.33712	0.130171	0.047092	0.822737
19	0.784734	0.390251	0.347377	0.087399	0.565224
20	0.448995	0.844184	0.010243	0.496448	0.49331
21	0.153055	0.066696	0.48058	0.407481	0.11194
22	0.133125	0.429296	0.371119	0.208363	0.420518
23	0.683508	0.763476	0.023812	0.481624	0.494564
24	0.549201	0.917055	0.160672	0.464937	0.374391
25	0.356724	0.256099	0.483048	0.466744	0.050208
26	0.327807	0.697749	0.027338	0.558872	0.41379
27	0.053689	0.12743	0.431545	0.38331	0.185145
28	0.640356	0.897235	0.758083	0.168258	0.073659

**Table 9:** Optimized Adaptive Reference Intervals.

Attribute	Optimized Adaptive Intervals
Skewness	[0.038 0.298 0.739 1.4 1.93 2.23 3.05]
Kurtosis	[9.87 15.4 22.4 38.6 61 89 146]
PeakFrequency	[35.6 2710 2820 2920 3230 3600 3850]
InterquartileRange	[0.116 0.13 0.14 0.146 0.154 0.161 0.185]

**Table 10:** Comparison with Different Data-Driven Methods.

	IBRB-HOAI	BPNN	KNN	DT	ELM	LR	SVM	CNN
AC	97.52%	93.14%	91.51%	92%	94.4%	92.31%	93.45%	94.71%
PC	97.31%	92.18%	90.23%	90.79%	92.91%	90.05%	92.65%	93.98%
RC	97.15%	92.1%	90.04%	91.39%	93.26%	89.75%	92.43%	94.01%
F1	97.25%	92.2%	90.22%	91.34%	93.53%	89.94%	92.91%	94.51%
MSE	0.002176	0.00503	0.04575	0.046881	0.089708	0.042914	0.0051987	0.0052123

**Table 11:** Comparison Table with Different IBRB Models.

	IBRB-HOAI	IBRB-PCMA	IBRB-PSO	IBRB-GA	IBRB-DE
AC	97.52%	91.49%	90.80%	90.51%	90.62%
PC	97.31%	90.81%	90.24%	89.95%	90.05%
RC	97.15%	90.53%	89.93%	89.61%	89.72%
F1	97.25%	90.64%	90.09%	89.84%	89.90%
MSE	0.002176	0.003771	0.004223	0.004651	0.004312

**Table 12:** Presents the average MSEs of different optimization algorithms obtained from 20 rounds of experiments.

MSE	IBRB-HOAI	IBRB-PCMA	IBRB-PSO
90%	0.002532	0.003245	0.003579
80%	0.002512	0.003392	0.003781
70%	0.002176	0.003771	0.004223
50%	0.002663	0.004124	0.004589
30%	0.002735	0.004217	0.004832

**Table 13:** Presents the average accuracy values of different optimization algorithms obtained from 20 rounds of experiments.

AC	IBRB-HOAI	IBRB-PCMA	IBRB-PSO
90%	96.73%	90.32%	89.23%
80%	97.14%	91.16%	90.15%
70%	97.52%	91.49%	90.80%
50%	96.21%	89.36%	88.62%
30%	95.87%	88.62%	88.41%

**Table 14:** Time Complexity Comparison.

Time Complexity	IBRB-HOAI	IBRB-PSO	IBRB-PCMA
Unit(s)	32.40634	34.47927	33.49235

**Table 15:** Fivefold cross-validation.

	Data 1	Data 2	Data 3	Data 4	Data 5
MSE	0.003639	0.001417	0.000798	0.001332	0.001664
AC	97.07407%	95.85185%	95.94444%	96.240745%	97.81481%

**Table 16:** MSE results over 20 independent repeated runs.

Number	IBRB-HOAI	IBRB	IBRB-PSO	IBRB-PCMA
1	0.001764	0.069415	0.00444	0.00573
2	0.001586	0.068262	0.004902	0.003171
3	0.000886	0.100955	0.001953	0.00545
4	0.001878	0.102433	0.005468	0.003402
5	0.002276	0.088285	0.004419	0.005102
6	0.002378	0.07024	0.005548	0.004038
7	0.003664	0.102792	0.005587	0.002665
8	0.002395	0.082749	0.003836	0.003236
9	0.002828	0.054743	0.004801	0.003056
10	0.001803	0.092192	0.005656	0.003969
11	0.002793	0.097246	0.003062	0.004165
12	0.002579	0.09114	0.003112	0.003376
13	0.002276	0.060483	0.005985	0.002763
14	0.002563	0.079407	0.005015	0.003832
15	0.00218	0.10533	0.002345	0.004157
16	0.002104	0.079535	0.006769	0.002833
17	0.001729	0.119599	0.004692	0.002679
18	0.002195	0.099344	0.00248	0.005153
19	0.001754	0.101136	0.002076	0.003428
20	0.001883	0.068125	0.002324	0.003212
Average	0.002176	0.086671	0.004223	0.003771

**Table 17:** Statistical significance analysis of MSE results using Wilcoxon signed-rank test with Bonferroni correction.

Comparison	Positive Rank Sum W+	Negative Rank Sum W−	Test Statistic W	Raw p-Value	Raw p-Value	Significance
IBRB-HOAI vs. IBRB	0	210	0	$1.9\times 10^{-6}$	$5.7\times 10^{-6}$	Significant
IBRB-HOAI vs. IBRB-PSO	0	190	0	$1.9\times 10^{-6}$	$5.7\times 10^{-6}$	Significant
IBRB-HOAI vs. IBRB-PCMA	4	206	4	$1.8\times 10^{-4}$	$5.4\times 10^{-4}$	Significant

## Data Availability

Due to privacy concerns, the authors do not have permission to share data.

## Appendix A

All variables are defined in Table A1.

**Table A1:** Dictionary of notations.

Notation	Meaning
$X={\left[x_{1},\cdots ,x_{S}\right]}^{T}$	The input attribute
S	The feature dimension
K(•)	A K-means clustering function
Q(•)	A quantile estimation function
$RI_{S}$	The adaptive reference interval for the sth feature
$H_{PSO-PCMAES}(•)$	The hybrid optimization operator
Φ	The initial parameter set
Ω	The set of algorithm hyperparameters
$\Phi_{best}$	The optimal BRB model parameter set
$C=\{C_{1},C_{2},\ldots C_{K}\}$	The partition of data into K disjoint clusters by K-means clustering
J(•)	The objective function of K-means clustering
$\zeta_{k}$	The centroid of cluster $C_{k}$
$C_{\mathrm{normal}}$	The selected reference cluster for the healthy state.
$\left|C_{k}\right|$	The number of samples in the kth cluster
$\arg\max_{k}$	The operator that finds the cluster k maximizing the objective
$L_{S}$	The lower bound of the reference interval
$U_{S}$	The upper bound of the reference interval
$X_{S}^{ref}$	The set of observed values of the sth feature extracted from the healthy cluster $C_{\mathrm{normal}}$
α	The significance level
$x_{i}$	The input premise attributes
$D_{i}$	The ith evaluation grade
$\beta_{i,k}$	The belief degree assigned to grade $D_{i}$ under the kth rule
N	The total number of evaluation grades
$\theta_{k}$	The rule weight of the kth rule
$\delta_{k}$	The rule reliability of the kth rule
$\sum_{i=1}^{N}\beta_{i,k}\leq 1\;$	The sum of belief degrees for each rule does not exceed unity
$a_{i}^{k}$	The matching degree between the ith input attribute and the kth rule
$x_{i}$	The actual input sample data for the ith attribute
$A_{i}^{l}$	The reference value of the ith attribute in the lth rule
$\Theta =\{D_{1},\ldots ,D_{N}\}$	the identification framework
$\beta_{n,i}$	The belief degree assigned to grade $D_{n}$ under the ith rule
$\beta_{\Theta ,i}$	The degree of global ignorance regarding the attribute of Θ
β(Θ)	The power set of the identification framework Θ
${\tilde{m}}_{n,i}$	The mixed probability mass of evidence i on evaluation grade $D_{n}$
${\tilde{m}}_{\beta (\Theta ),i}$	The mixed probability mass assigned to the entire power set
∅	The empty set
$c_{\gamma w,i}$	The normalization coefficient for the ith evidence
$m_{n,i}$	The weighted probability mass
${\hat{m}}_{n,e(k)}$	The unnormalized combined probability mass on grade $D_{n}$ after fusing the first k pieces of evidence
$m_{n,e(k-1)}$	The combined probability mass on grade $D_{n}$ after fusing the first k−1 pieces of evidence
$m_{\beta (\Theta ),e(k-1)}$	The combined probability mass on global ignorance after fusing the k−1 pieces of evidence.
$\beta_{n,e(k)}$	The final belief degree for evaluation grade $D_{n}$ after fusing the first k pieces of evidence.
L	The total number of pieces of evidence.
$\beta_{n,e(L)}$	The final belief degree for grade $D_{n}$ after fusing all L pieces of evidence.
$\beta_{\Theta ,e(L)}$	The global ignorance degree after fusing all evidence.
y	The final expected utility value
$u(D_{n})$	The utility value of evaluation grade $D_{n}$
u(Θ)	The utility value of global ignorance
min MSE(•)	The minimization of the mean squared error to align the estimated health state with the actual health state
$\beta_{n,k}^{*}$	The belief degree of the nth evaluation grade in the kth rule (optimization variable).
$\delta_{k}^{*}$	The reliability parameter of the kth rule (optimization variable).
$\theta_{k}^{*}$	the weight of the kth rule (optimization variable).
$y_{i}$	The estimated health state output of the model for the ith training sample
$y_{i}^{actual}$	The actual health state of the ith training sample
H	The total number of training samples
$v_{i}(t)$	The velocity of particle i at time t
$z_{i}(t)$	The position of particle i at time t
$pbest_{i}$	The personal best position of particle i
gbest	The global best position
w	The inertia weight
$q_{1}$	The individual experience factor
$q_{2}$	The social interaction factor
$r_{1}$ $\cdot r_{2}$	The random numbers
$ub_{i}$	The lower bound of particle i
$lb_{i}$	The upper bound of particle i
$v_{max}$	The maximum velocity limit
$f_{\mathrm{new}}$	The new fitness value (MSE)
$y_{i}(z_{i}(t+1))$	The model’s estimated output for the ith training sample under parameters $z_{i}(t+1)$
$z_{\mathrm{pso}}^{*}$	The optimal solution found by the PSO algorithm
$P_{\mathrm{pso}}$	The final PSO population
$z_{i}(T_{\mathrm{pso}})$	The position of particle i at the $T_{\mathrm{pso}}$ generation
$\Omega^{\left(0\right)}$	The initial mean vector of the P-CMA-ES algorithm
$\sigma^{\left(0\right)}$	The initial step size
ξ	The scaling factor
$N_{\mathrm{pso}}$	The total number of PSO particles
$x_{i}(T_{\mathrm{pso}})$	The position of particle i at the final generation
$\mu_{\mathrm{pso}}$	The mean of the final PSO population
||•||	The Euclidean norm
$C^{\left(0\right)}$	The initial covariance matrix
$P_{\sigma }^{\left(0\right)}$	The initial value of the step-size evolution path
$P_{c}^{\left(0\right)}$	The initial value of the covariance evolution path
$\Omega_{k}^{\left(g+1\right)}$	The kth solution at generation g+1
$mean^{\left(g\right)}$	The mean of the search distribution at generation g
$\varepsilon^{\left(g\right)}$	The step size factor at generation g
$C^{\left(g\right)}$	The covariance matrix at generation g
N(•)	The normal distribution
λ	The population size
$\beta_{k}^{\left(g+1\right)}$	The reasonable belief distribution
⇐	The replacement operation symbol
$A_{e}$	The equality constraint matrix
$n_{e}$	The number of constrained variables
${A_{e}}^{T}$	The transpose of the constraint matrix
$\psi_{k}$	The weight coefficient of the kth solution
τ	The number of selected offspring solutions
$p_{\sigma }^{\left(g\right)}$	The step-size evolution path at generation g
$c_{\sigma }$	The step-size path learning rate
$\mu_{\mathrm{eff}}$	The effective population size
$C^{{\left(g\right)}^{-\frac{1}{2}}}$	The square root of the covariance matrix
$\sigma^{\left(g\right)}$	The step size at generation g
$d_{\sigma }$	The step-size damping factor
$\left\|p_{\sigma }^{\left(g+1\right)}\right\|$	The Euclidean norm (length) of the updated step-size evolution path
$E\left[\left\|N(0,I)\right\|\right]$	The expected length of a standard normal random vector
$p_{c}^{\left(g\right)}$	The covariance evolution path at generation g
$c_{c}$	The covariance path learning rate
$\psi_{i}$	The weight coefficient of the ith solution
$h_{\sigma }^{\left(g+1\right)}$	The heuristic flag
$\chi_{D}$	The chi-distribution threshold
D	The search space dimension
$C^{\left(g\right)}$	The covariance matrix at generation g
$c_{1}$	The rank-1 learning rate
$c_{2}$	The rank-μ learning rate
$\omega_{i}$	The weight of the i-th solution
$\Omega_{k:\lambda }^{\left(g+1\right)}$	The kth good solution at generation g+1
$mean^{\left(G\right)}$	The mean of the search distribution at the final generation G
G	The total number of PCMA-ES iterations
AC	The proportion of correct predictions
NCP	The count of correctly predicted samples
TNP	The total number of prediction samples
PC	The proportion of true positives among predicted positives
TP	The correctly predicted positive samples
FP	The incorrectly predicted positive samples
RC	The proportion of true positives correctly identified
FN	The incorrectly predicted negative samples
